# Functionalized Nanoparticles Targeting Tumor-Associated Macrophages as Cancer Therapy

**DOI:** 10.3390/pharmaceutics13101670

**Published:** 2021-10-13

**Authors:** Yuanyuan He, Raimundo Fernandes de Araújo Júnior, Luis J. Cruz, Christina Eich

**Affiliations:** 1 Translational Nanobiomaterials and Imaging (TNI) Group, Department of Radiology, Leiden University Medical Center, 2333 ZA Leiden, The Netherlands; y.he@lumc.nl (Y.H.); fernandes.araujo@ufrn.br (R.F.d.A.J.); 2 Postgraduate Program in Health Science, Federal University of Rio Grande do Norte (UFRN), Natal 59064-720, Brazil; 3 Cancer and Inflammation Research Laboratory (LAICI), Postgraduate Program in Functional and Structural Biology, Department of Morphology, Federal University of Rio Grande do Norte (UFRN), Natal 59064-720, Brazil; 4 Percuros B.V., 2333 CL Leiden, The Netherlands

**Keywords:** tumor microenvironment, tumor targeting, nanoparticles, M1/M2 macrophages, cancer

## Abstract

The tumor microenvironment (TME) plays a central role in regulating antitumor immune responses. As an important part of the TME, alternatively activated type 2 (M2) macrophages drive the development of primary and secondary tumors by promoting tumor cell proliferation, tumor angiogenesis, extracellular matrix remodeling and overall immunosuppression. Immunotherapy approaches targeting tumor-associated macrophages (TAMs) in order to reduce the immunosuppressive state in the TME have received great attention. Although these methods hold great potential for the treatment of several cancers, they also face some limitations, such as the fast degradation rate of drugs and drug-induced cytotoxicity of organs and tissues. Nanomedicine formulations that prevent TAM signaling and recruitment to the TME or deplete M2 TAMs to reduce tumor growth and metastasis represent encouraging novel strategies in cancer therapy. They allow the specific delivery of antitumor drugs to the tumor area, thereby reducing side effects associated with systemic application. In this review, we give an overview of TAM biology and the current state of nanomedicines that target M2 macrophages in the course of cancer immunotherapy, with a specific focus on nanoparticles (NPs). We summarize how different types of NPs target M2 TAMs, and how the physicochemical properties of NPs (size, shape, charge and targeting ligands) influence NP uptake by TAMs in vitro and in vivo in the TME. Furthermore, we provide a comparative analysis of passive and active NP-based TAM-targeting strategies and discuss their therapeutic potential.

## 1. Introduction

In the past decades, cancer has been the leading cause of death worldwide. Although traditional therapies including surgery, radiotherapy and chemotherapy have reduced the incidence of cancer-related deaths, they lack selectivity towards tumor cells [1]. As a result, high doses of drugs are often needed for cancer treatment, leading to the occurrence of cytotoxicity and multi-drug resistance, while the effect on tumor metastasis and advanced tumors is poor. The immune system of treated cancer patients is often weakened, which increases the risk of recurrence and metastasis [2,3]. Thus, there is urgent need for more effective treatment strategies to fight cancer. More recently, exploring the relationship between the tumor and host cells in the TME has become a focus of research. As discussed in several recent reviews, unlimited proliferation, metastasis, immune and apoptosis escape mechanisms of cancer cells are closely related to the TME [4,5,6].

**Figure 1:**
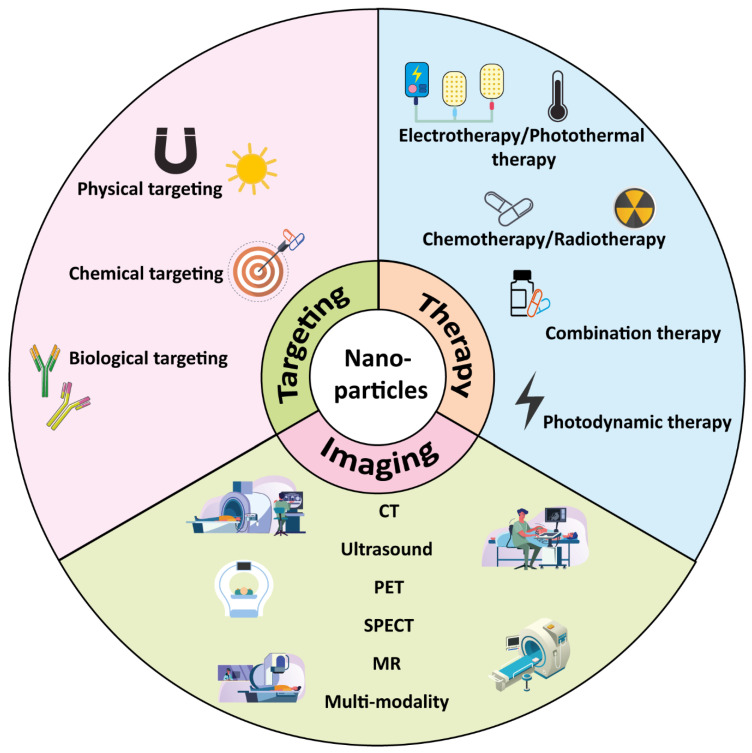
Applications of nanoparticles (NPs) in the field of biomedicine. NPs can be used for in vitro and in vivo targeted drug delivery, therapy and imaging. Multimodal imaging refers to the production of signals that can be detected by more than one imaging technique, for example, the combination of magnetic and radioactive substances to be detected by PET/MRI or PET/CT. Many NP formulations are multimodal and multifunctional. The latter refers to NPs that combine multiple objectives, such therapy and imaging. This figure was prepared using a template on the Servier medical art website (http://www.servier.fr/servier-medical-art, accessed on 10 June 2021).

In the past ten years, NP-based drug delivery systems with application in cancer therapy have aroused much interest [7,8,9]. According to the composition of NPs, NPs can be divided into metallic, organic, inorganic and polymeric nanostructures [10]. NPs can be utilized as delivery systems for different types of biomolecules, such as fluorophores, metals, peptides, proteins, oligonucleotides or biomimetic drugs, which gives NPs a multifunctional role in diagnosis and treatment. Controlled drug delivery by NPs can prevent premature drug degradation, improve drug absorption and reduce side effects. Depending on the NPs’ physicochemical properties, such as size, shape, charge and surface chemistry, as well as their optical properties, NPs have been widely used in tumor diagnostics and monitoring [11], drug delivery [12], optical imaging [13], development of synthetic vaccines [14] and the manufacturing of miniature medical devices [15] (Figure 1). In comparison to the bulk material, NPs possess new chemical and physical properties, can increase the biocompatibility and stability of conventional drugs and overcome problems of solubility. In addition, compared with traditional single-modality drugs, NPs can be designed to harbor multiple functions.

Most NPs are biodegradable and possess low cytotoxicity and low immunogenicity in vitro and in vivo [16]. In addition, the possibility to link targeting moieties can be utilized for site-specific delivery in vivo [17,18]. For example, the specific targeting of the BLP25 liposome vaccine to tumor cells mainly depends on the presence of an antibody on the liposome surface [19]. Biodegradable polymer materials with low cytotoxicity and high biocompatibility, such as polylactic acid (PLA), polyglycolic acid (PGA) and poly (lactic-co-glycolic acid) (PLGA), have been approved by the Food and Drug Administration (FDA) as drug delivery systems for vaccine antigens made of proteins, peptides and DNA [20,21]. PLGA is one of the most successfully developed polymers, and has been widely used in the encapsulation and targeted delivery of therapeutic agents to cancer cells to enhance the effect of anticancer therapy [22,23]. Compared to traditional therapies, NP-based therapeutics show longer circulation times in the bloodstream and encapsulated biological agents have longer half-lives than free drugs [24]. Polymeric NPs, such as those made of PLGA, typically show a sustained-release kinetics profile, which leads to uniform and prolonged drug levels in the body and reduced drug toxicity, compared to conventionally administered drugs [23,25]. In addition, PLGA NPs are often functionalized with polyethylene glycol (PEG), which improves the circulation time and half-life in the blood stream, and presents a stable linker to couple-targeting motifs for tumor-specific delivery [14,26,27].

To date, the FDA has approved more than 30 NP formulations for therapy or diagnostics, including metallic NPs, albumin NPs and liposomes, many of them with application in cancer therapy [20]. For example, Abraxane, an albumin-paclitaxel NP formulation, can passively target tumors through the enhanced permeability and retention (EPR) effect [28]. In addition, more than 50 NP formulations are currently in clinical trials and many more are in pre-clinical research [29].

In recent years, the TME has become a target of NP-based delivery systems aiming to prevent tumor progression and survival, to “re-educate” immunosuppressive M2 TAMs towards the M1 type with antitumor properties and to raise an antitumor immune response [7,8,9,30,31]. However, due to individual differences, tumor heterogeneity and complexity of the TME, different results have been reported [32]. Yet, targeting of TAMs in the TME in vivo could be key to the development of effective NP-based cancer treatments. In this review, we give an overview of TAM biology and refer to the latest research in the field of TAM-targeted immunotherapy. We will focus on strategies that utilize NPs to target and shape TAMs during tumor development and progression. In particular, we will pay special attention to how different types of TAMs interact with NPs, and how the physicochemical properties of NPs influence NP uptake.

## 2. Macrophages in Tumorigenesis

### 2.1. Tumor-Associated Macrophages (TAMs)

It is well known that the TME promotes tumorigenesis and tumor metastasis in addition to causing resistance to cancer therapy. Tumors are complex biological systems that primarily consist of cancer cells, vascular cells, mesenchymal lineage cells, immune cells of both lymphoid and myeloid origin and fibroblasts [33,34]. The interaction of these cell populations in tumors promotes the formation of the TME. TAMs represent one of the main tumor-infiltrating innate immune cells in the TME, playing a critical role in inflammation, which is well established to promote tumor growth and tumor progression.

**Figure 2:**
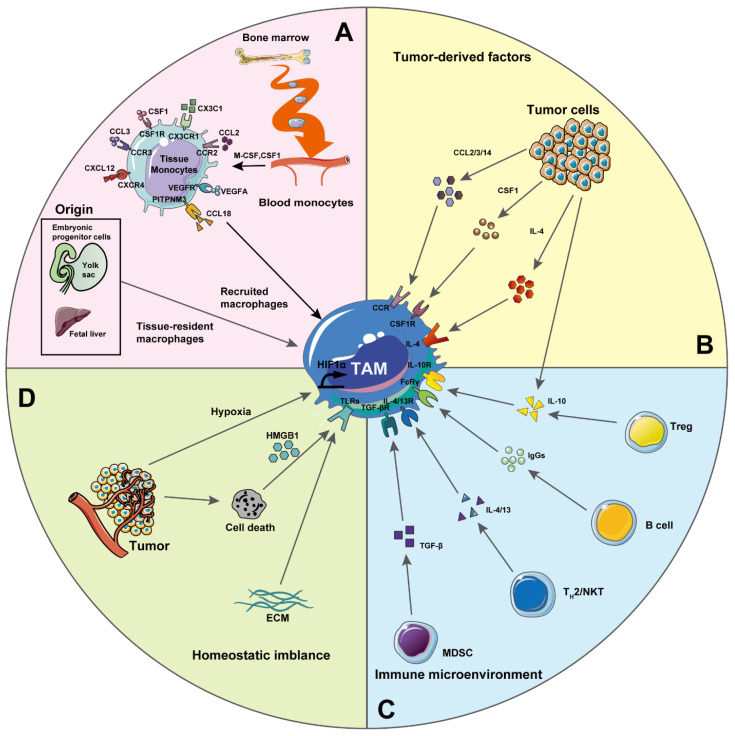
The origin and polarization state of tumor-associated macrophages (TAMs) in the tumor microenvironment (TME). (**A**) Origin: TAMs in the tumor are largely derived from blood monocytes that have their origin in hematopoietic stem cells in the bone marrow, or, at minor contributions, from locally derived, tissue-resident macrophages with their origin in progenitor cells generated in the fetal liver and yolk sac during embryonic development. (**B**) Tumor-derived factors: tumor cells can produce a variety of factors (such as CCL2/3/4, CSF1, IL-4 and IL-10) that act on the polarization of TAMs. (**C**) Immune microenvironment: Treg-derived IL-10, MDSC-derived TGFβ, B-cell-derived Ig, Th2-derived IL-4 and NK-cell-derived IL-13 enhance the M2-like (immunosuppressive) phenotype of TAMs. (**D**) Homeostatic imbalance: Hypoxia promotes the malignant transformation of tumors under the action of hypoxia-inducible factors. Tumor-derived ECM and HMGB1 increase the number of TAMs. The abbreviations are CSF1, colony-stimulating factor 1; CSF1R, CSF1 receptor; ECM, tumor-derived extracellular matrix; HMGB1, high-mobility group box 1 protein; Tregs, regulatory T cells; Ig, immunoglobulin; TGF, transforming growth factor; IL, interleukin; TNF, tumor necrosis factor; VEGF, vascular endothelial growth factor; VEGFR, VEGF receptor; MDSCs, myeloid suppressor cells; TLR, Toll-like receptor; CCL, chemotaxis chemokine ligand; CXCL, chemokine; and CXCR, chemokine receptor. This figure was prepared using a template on the Servier medical art website (http://www.servier.fr/servier-medical-art, accessed on 10 June 2021).

TAMs differ both from tissue-resident and bone-marrow-derived macrophages [34,35] (Figure 2). Although macrophages were originally described as arising exclusively from circulating monocyte precursors [36], more recently it was shown that several organs harbor embryonic-derived populations of resident macrophages that maintain and self-renew throughout adulthood [33,37,38]. These tissue-resident macrophages include brain microglia, alveolar, pancreatic, peritoneal, splenic, kidney and gut macrophages, as well as Langerhans and Kupffer cells [33,37] (Figure 2A). There are contradicting reports about the cellular origin of TAMs. For example, it has been reported that TAMs were recruited from CCR2+ inflammatory monocytes in a mouse model of mammary cancer [34]. However, in recent years, with the new concept of the embryonic origin of tissue-resident macrophages, it has been confirmed that both embryonic- and monocyte-derived macrophages constitute the pool of TAMs in the TME [39]. In models of murine pancreatic ductal adenocarcinoma, a large portion of tissue-resident macrophages was shown to originate from embryonic development, and to expand during tumor development [40]. The authors further reported functional heterogeneity among TAM subsets in the TME, which was correlated with their cellular origin. Monocyte-derived TAMs played more potent roles in antigen presentation, while embryonically derived TAMs exhibited a profibrotic transcriptional profile, indicative of their role in producing and remodeling molecules in the extracellular matrix during tumor progression. At the onset of tumor development, TAMs are mainly proinflammatory macrophages which promote tumor progression by inducing the expression of inflammatory cytokines and proangiogenic factors [39,40]. TAMs further support tumor malignancy and metastasis by enhancing the invasion and extravasation ability of tumor cells, inhibiting antigen presentation, as well as stimulating tumor relapse after chemotherapy [41,42,43].

### 2.2. Macrophage Polarization and Tumor Development

**Figure 3:**
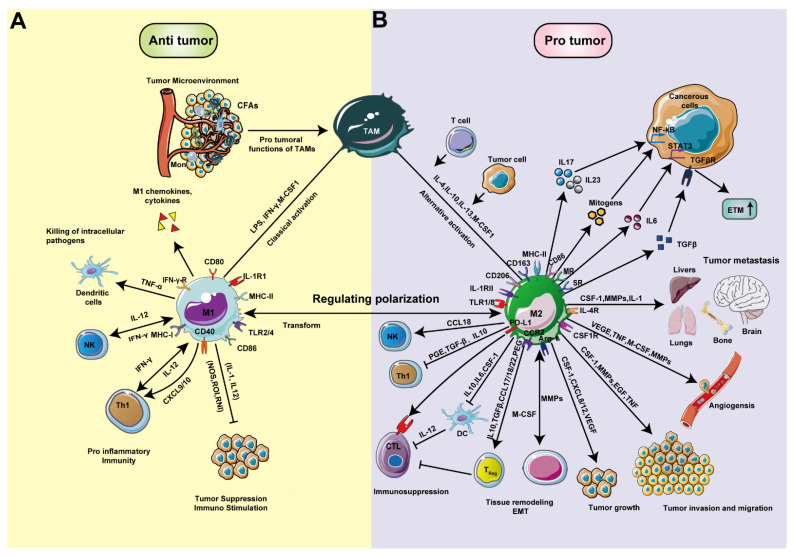
The role of tumor-associated macrophages (TAMs) in tumor progression. Macrophages are recruited into tumors under the action of chemokines and growth factors and gradually become TAMs. TAMs can be activated as (**A**) M1 macrophages with pro-inflammatory effects (antitumor) and (**B**) M2 macrophages with non-inflammatory properties (protumor). Among them, a variety of factors expressed and secreted by M2-like macrophages (such as VEGF, TNF-α, matrix metalloproteinase (MMP), CCL2 and CSF1) can induce tumor growth, migration, invasion, immunosuppression, treatment resistance and intravascular invasion. For example, M2 macrophages promote tumor migration and invasion through EGF, TNF, CSF-1 and MMPs; promote angiogenesis and tumor growth through the secretion of VEGF, MCSF, IL10 and TGF-β; promote immunosuppression through the secretion of CCL17/18/22, CSF -1, IL-10, TGF-β and PEG; promote tumor metastasis by secreting CSF-1, IL-1 and MMPs; and induce cancer cell proliferation through NF-κB or STAT3 signaling pathways. In contrast, the “classical activated” M1 macrophages are immune-stimulating, induce antitumor immunity and kill intracellular pathogens by the actions of IL-1, IL-6, IL-12, IL23, TNF-α, NOS, ROI and RNI. CTL, cytotoxic T lymphocytes; CAF, cancer-related fibroblasts; CSF1, colony-stimulating factor 1; TGF, transforming growth factor; IL, interleukin; TNF, tumor necrosis factor; MMP, matrix metalloproteinase; TLR, toll-type receptor; VEGF, vascular endothelial growth factor; LPS, lipopolysaccharide; NOS, inducible nitric oxide synthase; RNI, reactive nitrogen intermediates; and ROI, reactive oxygen intermediates. This figure was prepared using a template on the Servier medical art website (http://www.servier.fr/servier-medical-art, accessed on 10 July 2021).

Macrophages show remarkable functional plasticity and undergo specific differentiation in different tissue environments. Tissue-resident macrophages kill foreign pathogens and coordinate leukocyte infiltration and antitumor adaptive immunity through phagocytosis and the degradation of apoptotic cells, microorganisms and neoplastic cells [44]. However, cancer cells that have escaped the immune system slowly form an immunosuppressive TME, where the release of tumor-associated antigens as a consequence of tumor cell death is decreased and cancer cells successfully escape sanctions of the immune system [5]. TAMs are widely present in various tumors, participate in the formation of the TME and often exhibit a variety of polarization states under the influence of hormones, cytokines and regulatory molecules (Figure 3), which affect their role in innate and acquired immunity. In general, TLR and cytokine receptor signaling skews TAMs away from the “classically” activated tumoricidal phenotype (referred to as M1) towards an “alternatively” activated tumor-promoting one (M2) (Figure 3). M1 macrophage activation stimuli are interferon gamma (IFN-γ) and lipopolysaccharide (LPS), which induce strong microbicidal properties, production of the proinflammatory cytokines TNFα, type I interferons (IFNs), IL-1, CXCL1-3, IL-6 and IL-12 [45], and promote an IL-12-mediated Th1 response, which generates responses against intracellular parasites and neoplastic cells [46] (Figure 3A). In contrast, M2 macrophages are divided into type M2a, M2b and M2c, and the activation stimuli are IL-4, IL-10 or IL-13, immune complexes in combination with IL-1β or LPS, TGF-β or glucocorticoid stimulation [47]. A large number of M2-like TAMs reside in the TME and produce anti-inflammatory cytokines, pro-angiogenic and trophic factors, such as nitrate oxide and ornithine, and reduce the secretion of proinflammatory cytokines [48], which altogether have the ability to suppress antitumor immune responses [49], and promote tumor neovascularization as well as tumor growth [50] (Figure 3B).

The differential expression of various TLRs on macrophages is an important factor underlying macrophage polarization [51]. Mechanistically, macrophage polarization is regulated by interferon regulatory factors (IRFs) and the signal transducer and activator of transcription proteins (STAT) signaling pathway [52]. For example, IFN and TLR signals promote the polarization of macrophages towards the M1 type by activating the IRF/STAT1 signaling pathway [53]. Macrophage proliferation is a significant feature of advanced stages of cancer. Giurisato et al. demonstrated that a high proportion of proliferating macrophages in human tumors expresses extracellular-regulated protein kinase 5 (ERK5) [54,55]. The authors showed that ERK5 supported the proliferation of macrophages in melanoma tumor grafts in mice by suppressing p21 expression [56], which led to the halt of the macrophage differentiation program. Inhibition of ERK5 blocked macrophage polarization and reduced metastasis in vivo [57]. In another study, the authors demonstrated that ERK5-deficient TAMs displayed a proinflammatory phenotype. Mechanistically, ERK5 inactivation suppressed the phosphorylation of STAT3, thereby inhibiting the proliferation of melanoma and carcinoma grafts [57]. In contrast, IL-4- and IL-13-induced signaling pathways promote macrophages to polarize towards the M2 phenotype via STAT6 [58]. Apoptotic tumor cells promote macrophage polarization towards the M2 type via the phosphatidylinositol 3-kinase (PI3K)/protein kinase B (Akt) signaling pathway [59]. Activation of the Akt signaling pathway has also been shown to reduce tumorigenesis. Given that Akt subtypes (Akt1 and Akt2) play opposite roles in macrophage polarization, antitumor responses can be adjusted by the activation of different Akt subtypes [60] (Figure 3B). Importantly, M1 and M2 polarization are dynamic processes and the phenotypes of polarized macrophages can be reversed under different physiological and pathological conditions [61]. However, the molecular mechanisms that control the phenotype of macrophages are not fully understood yet.

TAMs resemble the M2 phenotype and play a harmful tumor-promoting role. When cancer occurs, IL-10 and TGF-β secreted by TAMs can prevent an antitumor immune response by inhibiting the function of antigen-presenting cells and effector T cells, leading to the escape of cancer cells and the formation of an immunosuppressive TME [62]. The multifunctional cytokine IL-10 plays a vital role in supporting tumorigenesis and inhibiting the activation of monocytes, T cells and macrophages required for antitumor immunity [63]. Studies in mouse tumor models showed that IL-10 not only inhibits the production of IL-12, but also inhibits the maturation of dendritic cells and limits the cytotoxic T cell response during chemotherapy [64]. Regulatory molecules released by Treg cells, such as IL-35, IL-10, adenosine and TGF-β, inhibit Th1, Th12 and Th17 immune responses, which promote antitumor functions [33,62,65,66]. IFN-γ produced by Th1 cells can up-regulate histocompatibility complex (MHC) class I molecules. The loss or down-regulation of MHC class I molecules on the surface of tumor cells is a tumor immune escape mechanism. Yoo et al. showed that defects in MHC class I molecules cause a poor prognosis in PD-L1-positive head and neck squamous cell carcinoma [67]. In addition, the interaction of PD-1/PD-L1 has been shown to suppress antitumor immunity. For example, Wang et al. showed that the intrinsic PD-1/PD-L1 axis of tumor cells not only inhibits the Akt and extracellular-signal-regulated kinase (ERK)1/2 signaling pathways and tumor growth but also prevents interaction with T cells [68]. In summary, although immune cells, such as T cells, can inhibit tumor growth, their function is inhibited in many cancers. Therefore, how to rationally and effectively reactivate the immune system to kill tumors remains a major challenge.

## 3. Macrophages in Tumor Therapy

T. Honjo and JP Allison won the Nobel prize in physiology and medicine in 2018 for the discovery of inhibition of negative immune regulation as a form of cancer therapy [69]. Compared to traditional therapies, immunomodulation has great potential in cancer treatment.

### 3.1. Tumor Therapy Targeting Tumor-Associated Macrophages (TAMs)

**Figure 4:**
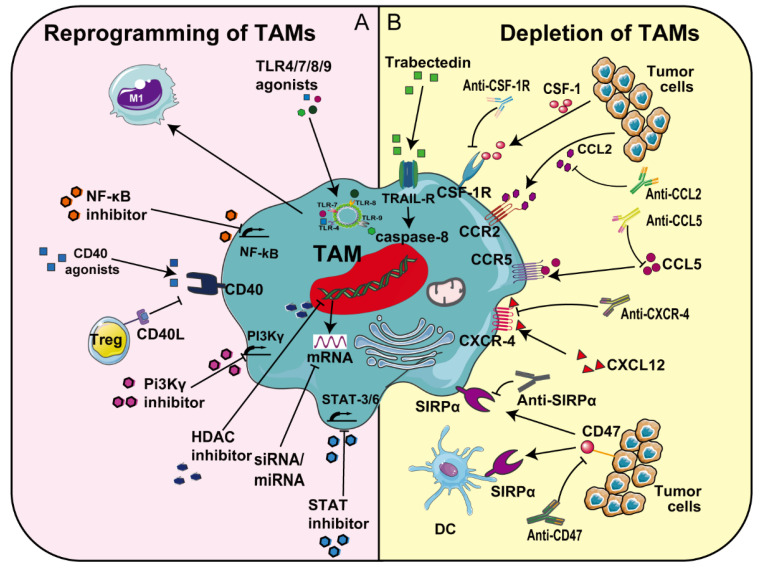
The main strategies for targeting tumor-associated macrophages (TAMs) in cancer therapy. (**A**) TAM reprogramming is mainly achieved by modulating signaling pathways using agonists of TLRs and CD40, inhibitors of NF-κB, PI3Kγ, STAT and HDAC, and si/miRNA. (**B**) Depletion of TAMs. TAM depletion strategies focus on using anti-neoplastic drugs, such trabectedin, and monoclonal antibodies that block signaling axes, including CCL2/CCR2, CSF-1/CSF-1R, CXCL12/CXCR4, or the interaction of SIRP1α-CD47. This figure was prepared using a template on the Servier medical art website (http://www.servier.fr/servier-medical-art, accessed on 10 July 2021).

In the TME, due to the complex relationship between cancer cells and surrounding immune cells, single-modality cancer treatments are often ineffective. Recruitment of TAMs to the TME has been shown to be associated with resistance to immune-mediated tumor cell killing, and the inhibition of their recruitment or differentiation prevented tumor growth [70] (Figure 4). At present, targeting macrophages has become a focus in the development of current cancer treatments and much clinical and preclinical research is under way. TAM-targeting strategies for cancer therapy can be divided into two main approaches: depletion of TAMs (i.e., by preventing their recruitment) and reprogramming of TAMs.

Cancer cells secrete a variety of cytokines and chemokines to recruit monocytes to the tumor side, including IL-6, IL-8, IL-34, CCL2, CCL3, CCL5, CCL20, CSF-1, CSF-2 and C-X-C motif chemokine 12 (CXCL12). In addition, other cells of the TME can also secrete cytokines/chemokines to recruit monocytes. For example, IL-8 secreted by cancer-associated fibroblasts (CAFs), IL-17 secreted by Th17 cells and CCL2 secreted by mesenchymal stem cells (MSCs). Major targets of current strategies to prevent monocyte recruitment and polarization towards the immunosuppressive M2 state are the CSF-1-CSF-1R, CCL2-CCR2, CXCL12-CXCR4 and CCL5-CCR5 signaling axis [71].

#### 3.1.1. CSF-1/IL-34-CSF-1R

CSF-1R on the surface of TAMs can combine with the ligands CSF-1 and IL-34 [72]. CSF-1 participates in the recruitment of TAMs to tumor tissues and the M2 polarization process of TAMs. The survival, proliferation and function of TAMs depend to a large extent on CSF-1R signaling (Figure 4). Blocking the CSF-1-CSF-1R axis inhibits the polarization of macrophages, thereby reducing tumor cell proliferation and inducing TAM apoptosis [73] (Figure 4). Many preclinical studies demonstrated high efficacies of CSF-1R inhibitors. For example, using mouse glioblastoma as an experimental object, researchers used CSF-1R inhibitors to reduce the expression of *Arg1* (arginase 1) and *Mrc1* (CD206, mannose receptor) genes in the TME, thereby inhibiting tumor growth and metastasis [74]. CSF-1R inhibitors, such as PLX6134 and GW2580, increased the CD8+ T cell response by inhibiting the CSF-1-CSF-1R signaling axis, eventually leading to TAM apoptosis [75]. Many current clinical treatment strategies targeting the CSF-1/CSF-1R pathway have been extensively developed [76], as summarized in Table 1. For example, in ongoing clinical trials targeting CSF-1, anti-CSF-1R monoclonal antibodies (such as IMC-CS4 in NCT01346358) block the binding of CSF-1 and IL-34 to CSF-1R and abrogate the recruitment and survival of TAMs, leading to TAM apoptosis and inhibition of tumor growth [77]. FPA008, an anti-CSF-1R antibody, is used in several clinical trials to treat advanced cancers, including lung, head, neck, pancreatic and ovarian cancer, as well as renal cell carcinoma and malignant glial tumor (i.e., NCT02526017 and NCT02471716) (Table 1). The first phase of the study, the dose-ranging phase, was well-tolerated by patients. However, current clinical studies have so far not shown that CSF-1R inhibitors, as a monotherapy, delay tumor growth. Combinational therapies of CSF-1R and CXCR2 antagonist demonstrated stronger effects on tumor treatment [78].

#### 3.1.2. CCL2-CCR2

CCL2 secreted by tumor cells can attract Th1 cells, Th17 cells and macrophages, which express high levels of CCR2, to the tumor site [79]. The CCL2-CCR2 axis has been shown to recruit TAMs for the construction and maintenance of an immunosuppressive TME [80,81]. Inhibition of the CCL2–CCR2 interaction can significantly reduce the number of TAMs in the tumor, thereby inhibiting tumor growth and spread [82]. In addition, the CCL2-CCR2 signaling pathway regulates macrophage polarization during cancer treatment. In a mouse model of non-small-cell lung cancer (NSCLC), it has been shown that estrogen receptor alpha (Erα) can activate the CCL2-CCR2 axis to promote macrophage infiltration, M2 polarization and MMP9 production, which increased NSCLC cell invasion [83]. Mechanistically, ERα was shown to bind to estrogen response elements on the CCL2 promoter, thereby increasing CCL2 expression. Using anti-estrogens or CCR2 antagonists to target the CCL2-CCR2 axis may improve outcomes in NSCLC [83]. In a murine breast tumor model, targeting the CCL2-CCR2 axis by complexes of CCR2 siRNAs and TAT cell penetrating peptides enhanced the efficacy of immunotherapy and promoted the reprogramming of TAMs [84]. Preclinical studies of hepatocellular carcinoma have shown that activated hepatic stellate cells recruit macrophages through the CCL2-CCR2 pathway and have the ability to induce M2 phenotypic transformation [85]. In clinical studies, Carlumab (anti-CCL2 monoclonal antibody) was proven to effectively resist the metastasis of prostate cancer by inhibiting the CCL2-CCR2 signaling pathway (NCT00992186) (Table 1). As a CCR2 antagonist, PF-04136309 has been well-tolerated in clinical studies of metastatic pancreatic ductal adenocarcinoma (NCT02732938) (Table 1). The combination of PF-04136309 and the chemotherapeutic drugs nab-paclitaxel/gemcitabine promoted the reduction in TAMs in tumors [86]. Therefore, the CCL2-CCR2 signaling pathway represents a promising target in the process of cancer treatment.

#### 3.1.3. CCL5-CCR5

In the TME, high concentrations of CCL5 can promote the accumulation of immunosuppressive cells and promote immune tolerance. Conversely, CCL5 can also recruit immune effector cells, such as lymphocytes, with high CCR5 expression (Figure 4). Phyllodes breast tumors promoted malignant progression by secreting CCL5 to recruit macrophages, which subsequently turned into TAMs [87]. In preclinical studies using mouse models of ovarian cancer, osteosarcoma, prostate and breast cancer, CCL5 antagonists inhibited cancer cell migration and invasion [88,89,90,91]. Mechanistically, crosstalk between CCR5 signaling and MMP-9 secretion has been reported. Anti-CCL5 blocking antibody or CCL5-shRNA knockdown in ovarian cancer stem-like cells decreased MMP-9 mRNA expression, and consequently reduced invasiveness [91]. Recently, the results of phase 2 clinical trials of CCR5 antagonist maraviroc in hematological malignancies and Hodgkin’s lymphoma showed that not only was the accumulation of monocytes was inhibited, but the migration of cancer cells was also effectively reduced (NCT01785810) (Table 1) [92]. Additionally, expression of the Raf kinase inhibitor protein (RKIP) can regulate TAM recruitment by blocking high-mobility group AT-hook 2 (HMGA2), leading to decreased expression of CCL5 [93]. This shows that targeted recovery of RKIP expression to reduce the secretion of CCL5 in tumor cells might be an important strategy to reduce macrophage infiltration [93].

#### 3.1.4. CXCL12-CXCR4

The CXCR4-CXCL12 axis recruits and keeps TAMs in the tumor’s hypoxic area and contributes to neo-angiogenesis and oxygen delivery [94,95] (Figure 4). Activation of chemokine receptor CXCR4/CXCL12 signaling has been shown to be implicated in TAM recruitment in melanoma [96]. Moreover, high expression of CXCL4 was intimately related to the invasion potential of TAMs in tumor tissues and the development of metastasis in NSCLC [97]. Studies have found that blocking CXCL12 can activate the MAPK/PI3K/AP-1 signaling pathway in colon cancer cells, thereby blocking the growth of tumor cells [98]. Blocking the interaction of CXCL12 with its receptor, CXCR4, is also thought to regulate macrophage infiltration and prevent metastasis. For example, the combination of anti-CXCR4 and anti-PD-1 immunotherapy to regulate TAMs in the microenvironment of glioma cells not only promoted an antitumor immune response, but also increased the level of inflammatory cytokines [99] (Figure 4). This strategy is currently employed in preclinical models, including metastatic pancreatic ductal adenocarcinoma, pancreatic neoplasms and prostate cancer [96,100]. CXCR4 antagonist BL-8040 combined with PD-1 blockers in a phase 2 trial of pancreatic ductal adenocarcinoma enhanced the efficacy of chemotherapy (NCT02826486) (Table 1) [101].

#### 3.1.5. CD47-SIRPα

The “do not eat me” signal CD47, overexpressed on the surface of cancer cells, forms a signaling complex with signal-regulatory protein α (SIRPα), a myeloid-specific immune checkpoint, enabling the escape from macrophage-mediated phagocytosis [102]. Therefore, inhibiting CD47-SIRPα signal transduction promotes the phagocytosis of tumor cells by macrophages and inhibits tumor growth [103]. Some preclinical studies have shown that in tumor models, anti-CD47 antibody treatment not only promoted the phagocytosis of cancer cells by macrophages, but also induced an antitumor cytotoxic T cell immune response [104]. In a mouse model of lymphoma, the bispecific anti-CD47/SIRPα antibody inhibited tumor growth and spread by targeting the multiple myeloid cell types in the tumor [105]. Anti-MUC1 and anti-EGFR (cetuximab) antibodies have achieved effective antitumor effects in carcinoma A549 cancer cells, causing tumor regression by inhibiting the binding of SIRPα and CD47 [106] (Figure 4). Currently, several clinical antitumor therapies targeting the CD47-SIRPα axis have been developed, such as two anti-CD47 monoclonal antibodies (Hu5F9-G4 and CC-90002), a soluble recombinant SIRPα-crystallizable fragment (Fc) fusion protein (TTI-621) [107,108] (i.e., NCT02216409 and NCT02678338) (Table 1). For example, in phase I clinical trials of advanced solid tumors and blood cancers, CC-90002, an anti-CD47 antibody, is used to block over-expressed CD47 on cancer cells, thereby inhibiting the CD47-SIRPα signaling pathway and enhancing the phagocytosis of tumor cells by macrophages [102].

In the TME, re-education of TAMs into M1-like macrophages has gradually become a more effective cancer treatment strategy. CD40 is expressed on dendritic cells, B cells, macrophages and even tumor cells (Figure 4). Under normal conditions, a CD40 ligand (CD40L and CD154) on activated T helper cells interacts with antigen-presenting cells via CD40, resulting in CD8+ T cell activation [109]. Tumor cells can undergo apoptosis or growth inhibition after CD40 ligation [27]. Multiple agonistic CD40 antibodies have been developed for clinical use. Currently, a variety of CD40-targeting agonist monoclonal antibodies are in phase 1 in clinical trials, including CDX-1140 and CDX-301 [110] for the treatment of advanced malignant tumors and solid tumors (Table 1). Responses in melanoma and pancreatic cancer have been reported [111]. Interestingly, blocking CD40 also promotes the polarization of TAMs to M1-like macrophages via the production of IL-12 [109].

#### 3.1.6. PI-3 Kinase γ (PI3Kγ)

*PI-3 kinase γ (PI3Kγ),* which is highly expressed in immune cells, such as mast cells, neutrophils and eosinophils, is a promising target for stimulating TAM re-polarization (Figure 4). The PI3Kγ signaling pathway activated by Akt and mTOR has the ability to stimulate C/EBPβ activation and inhibit the activation of NF-κB, which not only promotes TAM repolarization, but also inhibits tumor growth [112]. For example, in an animal model of pancreatic ductal adenocarcinoma, inhibiting the activity of PI3Kγ can reprogram TAMs and stimulate CD8 + T cells, thereby inhibiting tumor cell migration [110]. Signal pathway inhibitors for PI3Kγ have also demonstrated anti-metastatic effects in clinical tumor research. For example, PI3K/mTOR inhibitor PF-05212384 (Table 1) enhanced the antitumor effect and reduced tumor metastasis in head and neck squamous cell carcinoma in vivo [113].

Small molecule drugs, such as TLR agonists and cytokines, have attracted much attention in cancer therapy. TLR-mediated signaling pathways can repolarize M2 macrophages into the M1 type during tumor treatment to enhance antitumor immunity. For example, Resimod (TLR agonist), which targets TLR7/8, re-educates TAMs towards the M1 type in human colon carcinoma, while inhibiting tumor growth [114]. Cytokines, such as IL-6, IL-17 and IL-23 produced by the NF-κB or STAT3 signaling pathway, induce cancer proliferation and metastasis by promoting TAMs to inhibit a cytotoxic T cell response [115] (Figure 4). Therefore, NF-κB and STAT3 are also targets for drug therapy. NF-κB/STAT inhibitors can regulate the polarization of macrophages and inhibit tumor growth and metastasis [116,117]. Interestingly, the natural lipids ceramide and palmitic acid were recently shown to be effective inhibitors of the NF-κB or STAT3 signaling pathway, which inhibited the migration capacity of colorectal cancer cells, and at the same time promoted the repolarization of M2 TAMs towards the M1 type [118]. An ongoing clinical trial explores the STAT3/NF-κB/polycytosine kinase inhibitor IMX-110 in combination with low-dose doxorubicin (NCT03382340) to kill cancer cells (Table 1). In this context, NPs are used as drug carriers to deliver IMX-110 to the TME. Such nano-drug delivery systems proved to be advantageous in delivering drugs to the TME and TAMs, compared to systemically administered naked drugs [119].

Class IIa histone deacetylase (HDAC) has the property of regulating the antitumor immunity of macrophages in tumors (Figure 4A). Studies by Lobera et al. have demonstrated that class IIa HDAC inhibitors can promote macrophages to favor the proinflammatory M1 phenotype in the TME [120]. Similarly, research by Li et al. in mouse tumor models showed that HDAC inhibitors can not only reduce the migration of MDSCs to tumor sites, but also enhance the expression of antitumor phenotypes and the activation of T cells [49]. Furthermore, in the breast cancer model of MMTV-PyMT transgenic mice, Guerriero et al. found that class IIa HDAC inhibitors (TMP195) recruit and differentiate macrophages as well as promote their conversion into hyperphagocytic macrophages, thereby reducing tumor burden and tumor lung metastasis [121]. Therefore, the use of class IIa HDAC inhibitors to extract the potential of antitumor function in TAMs is a potential means of cancer treatment.

The crosstalk between tumor cells and macrophages is affected by microRNAs (miRNAs). Tumor-derived factor miRNAs, a kind of small non-coding RNA, can regulate target gene expression after transcription [122] (Figure 4A). According to reports, miRNA dysregulation can induce the occurrence of a variety of malignant tumors, and it has a significant impact on tumor immune escape, invasion, angiogenesis, migration and drug resistance [123,124]. For example, Zhao et al. found through studies on hepatocellular carcinoma specimens that miRNA-144/miRNA-451a promotes the polarization of TAMs to M1 macrophages by targeting hepatocyte growth factor and macrophage migration inhibitory factor [125]. In addition, small interfering RNAs (siRNA) can also target TAMs and modify the polarization and function of M2 TAMs by silencing the corresponding target gene [126] (Figure 4A). For example, Song et al. silenced endothelial growth factor and placental growth factor by delivering VEGF siRNA/PIGF siRNA to M2-type macrophages and breast tumor cells in vivo. The results showed that M2-type macrophages were re-educated towards M1-type macrophages, and the proliferation and migration of breast cancer cells were inhibited [127].

**Table 1:** Clinical trials of agents that target TAMs for cancer treatment.

Action	NCT Number	Tumor Type	Status	Target	Agent Name	Phase	Effect
Targeting signal transduction pathways	NCT02404844	Breast cancer	Completed	PI3K pathway	BKM120 Tamoxifen	Phase 2	Well-tolerated, preliminary activity in advanced cancers [128,129]
	NCT02384239	Breast cancer	Active, not recruiting	PI3K pathway	Palbociclib	Phase 2	NA
	NCT02058381	Breast cancer	Completed	PI3K pathway	Alpelisib (BYL719) Buparlisib (BKM120)	Phase 1b	Well-tolerated, preliminary antitumor activity [130]
	NCT01298713	Breast neoplasms	Completed	PI3K-Akt- mTOR pathway	Tamoxifen Everolimus	Phase 2	Well-tolerated, positive correlation [131]
	NCT01971515	Solid tumor	Completed	p70S6K/Akt	MSC2363318A	Phase 1	NA
	NCT02322853	Metastatic breast cancer	Terminated	MAK pathway	Tamoxifen, ralimetinib (LY2228820)	Phase 2	NA
	NCT03458221	Ovarian carcinoma	Not yet recruiting	ST pathway	Itraconazole	Phase 3	NA
	NCT00189358	Ovarian cancer, cancer of the fallopian tube and peritoneal cancer	Completed	the EGFR pathway	ZD1839 Tamoxifen	Phase 2	No side effects Ineffective for refractory/drug resistant cancers [132]
	NCT02040857	Breast cancer	Active, not recruiting	CDK 4/6	Palbociclib	Phase 2	Well-tolerated [133]
	NCT01920061	Neoplasm	Completed	PI3K/mTOR pathway	PF-05212384 (gedatolisib)	Phase 1	NA
	NCT02228681	Endometrial cancer	Active, not recruiting	mTOR pathway	Tamoxifen, everolimus, letrozole and medroxyprogesterone acetate	Phase 2	Enhanced anticancer efficacy
	NCT04318223	Metastatic breast cancer and locally advanced breast cancer	Recruiting	CDK4/6	Palbociclib	Phase 2	NA
	NCT02868268	Neuroblastoma	Recruiting	PD-1/PD-L1 pathway	Gene panel sequencing of tumor specimens	Phase NA	NA
	NCT03382340	Pancreatic cancer, breast cancer and ovarian cancer	Recruiting	STAT3/NF-kB	Imx-110	Phase 1, 2	NA
	NCT04504552	Oral premalignant lesions	Not yet recruiting	PD-1/PD-L1 pathway	Avelumab	Phase 2	NA
TAM recruitment	NCT02471716	Pigmented villonodular synovitis and tenosynovial giant-cell tumor	Completed	CSF-1R	FPA008	Phase 1, 2	NA
	NCT02526017	Advanced solid tumors, not limited to lung cancer, head and neck cancer, pancreatic cancer and ovarian cancer	Completed	CSF-1R	FPA008	Phase 1, 2	NA
	NCT02323191	Solid tumor	Completed	CSF-1	RO5509554	Phase 1	NA
	NCT02760797	Neoplasms	Completed	CSF-1	RO5509554	Phase 1	NA
	NCT03153410	Pancreatic cancer	Recruiting	CSF-1	IMC-CS4	Early phase 1	NA
	NCT04066244	Amyotrophic lateral sclerosis	Recruiting	CSF-1	BLZ945	Phase 2	NA
	NCT02829723	Advanced solid tumors	Recruiting	CSF-1	BLZ945	Phase 1, 2	NA
	NCT02880371	Advanced solid tumors	Completed	CSF-1	ARRY-382	Phase 1, 2	NA
	NCT01316822	Metastatic cancer	Completed	CSF-1	ARRY-382	Phase 1	NA
	NCT02777710	Colorectal cancer, pancreatic cancer, metastatic cancer and advanced cancer	Completed	CSF-1	PLX3397	Phase 1	NA
	NCT02584647	Sarcoma and malignant peripheral nerve sheath tumors	Recruiting	CSF-1	PLX3397	Phase 1	NA
	NCT02371369	Pigmented villonodular synovitis, giant-cell tumors of the tendon sheath and tenosynovial giant-cell tumor	Active, not recruiting	CSF-1	PLX3397	Phase 3	Mild side effects, improved patient symptoms and functional outcome [134,135,136,137]
	NCT01804530	Solid tumor	Terminated	CSF-1	PLX7486 TsOH	Phase 1	NA
	NCT03320330	Recurrent malignant solid neoplasm and osteosarcoma Refractory malignant solid neoplasm and osteosarcoma	Active, not recruiting	SEMA4D or CD100	Pepinemab (VX15/2503)	Phase 1 Phase 2	NA
	NCT03557970	Recurrent acute myeloid leukemia and refractory acute myeloid leukemia	Active, not recruiting	CSF-1	JNJ-40346527	Phase 2	NA
	NCT03690986	Squamous cell carcinoma of the head and neck	Recruiting	Recruiting	VX15/2503 (ipilimumab) Nivolumab	Phase 1	NA
	NCT03769155	Pathologic stage IIIB/C/D cutaneous melanoma AJCC v8	Recruiting	Recruiting	VX15/2503 (pepinemab)	Phase 1	NA
	NCT03177460	Prostate adenocarcinoma	Active, not recruiting	CSF-1	JNJ-40346527	Phase1	NA
	NCT01572519	Relapsed or refractory Hodgkin lymphoma	Completed	CSF-1	JNJ-40346527	Phase 1	NA
	NCT01597739	Arthritis, rheumatoid	Completed	CSF-1	JNJ-40346527	Phase 2	NA
	NCT02732938	Metastatic pancreatic ductal adenocarcinoma	Terminated	CCR-2	PF-04136309	Phase 2	Safe and tolerable [86]
	NCT01413022	Pancreatic neoplasms	Completed	CCR-2	PF-04136309	Phase 1	NA
	NCT01785810	Hematologic malignancy	Completed	CCR5	Maraviroc	Phase 2	NA
	NCT02826486	Metastatic pancreatic adenocarcinoma	Active, not recruiting	CXCR4	BL-8040	Phase 2	Safety, efficacy and immunobiological effects [101]
	NCT00992186	Prostate cancer	Completed	CCL2	Carlumab	Phase 2	NA
	NCT01494688	Solid tumor	Completed	CSF1-R	RG7155	Phase 1	NA
Targeting TAM activation	NCT04331067	Triple-negative breast cancer	Not yet recruiting	TIL and TAM	Paclitaxel, carboplatin Nivolumab, cabiralizumab	Phase 1, 2	NA
	NCT03285607	Breast cancer	Withdrawn	CSF-1	MCS110, cyclophosphamide and paclitaxel	Phase 1	NA
	NCT02435680	Triple-negative breast cancer	Completed	CSF-1	MCS110, carboplatin and gemcitabine	Phase 2	NA
	NCT01643850	Giant-cell tumor of the tendon sheath	Completed	CSF-1	MCS110 Placebo	Phase 2	NA
	NCT00757757	Prostate cancer and bone metastases	Terminated	CSF-1	MCS110	Phase 1, 2	NA
	NCT01346358	Neoplasms	Completed	CSF-1R	IMC-CS4	Phase 1	NA
	NCT03153410	Pancreatic cancer	Recruiting	CSF1-R	IMC-CS4	Phase 1	NA
	NCT01309230	Ovarian cancer	Active, not recruiting	Bi-shRNA furin and GMCSF	Vigil™	Phase 2	NA
	NCT02390752	Neurofibroma, plexiform, precursor cell lymphoblastic, leukemia–lymphoma, leukemia and acute sarcoma	Recruiting		PLX3397	Phase 1, 2	NA
	NCT04079712	Metastatic large-cell neuroendocrine carcinoma, metastatic neuroendocrine carcinoma, metastatic neuroendocrine neoplasm and metastatic small-cell carcinoma	Recruiting	PD-L1	Cabozantinib Cabozantinib S-malate Ipilimumab Nivolumab	Phase 2	NA
	NCT02265536	Neoplasms and neoplasm metastasis	Completed		LY3022855	Phase 1	NA
	NCT04550624	Advanced cholangiocarcinoma	Not yet recruiting		Pembrolizumab injection Lenvatinib mesylate	Phase 2	NA
	NCT01444404	Advanced malignancy, advanced solid tumors, cancer, oncology and tumors	Completed	CSF-1R	AMG 820	Phase 1	NA
	NCT02223312	Cancer Hematologic malignancies	Withdrawn	DC	TAPA-pulsed DC vaccine	Phase 1, 2	NA
	NCT01217229	Hodgkin lymphoma	Completed		PLX3397	Phase 2	NA
	NCT01804530	Solid tumor, tenosynovial giant-cell tumor	Terminated	CSF-1R	PLX7486 TsOH	Phase 1	NA
	NCT01596751	Metastatic breast cancer	Completed	DC	PLX3397 Eribulin	Phase 1, 2	NA
	NCT00637390	Ovarian cancer, fallopian tube cancer and peritoneal cancer	Terminated	CD52	Alemtuzumab	Phase 1	NA
	NCT01525602	Solid tumors	Completed		PLX3397 Paclitaxel	Phase 1b	NA
Reprogramming TAMs	NCT03285607	Breast cancer	Withdrawn	CSF-1	MCS110, doxorubicin, cyclophosphamide and paclitaxel	Phase 1	NA
	NCT00492167	Neuroblastoma	Active, not recruiting		Beta-glucan monoclonal antibody 3F8	Phase 1	NA
	NCT03954691	Cancer of head and neck	Not yet recruiting	Microglia, macrophages and NK cells.	Brain-tumor-immune cell communication	NA	NA
	NCT02953782	Colorectal neoplasms Solid tumors	Completed	CD47	Hu5F9-G4	Phase 1, 2	NA
	NCT03558139	Solid tumor and ovarian cancer	Active, not recruiting	CD47	Hu5F9-G4	Phase 1	NA
	NCT02216409	Solid tumor	Completed	CD47	Hu5F9-G4		Safe and tolerable [108,138]
	NCT01561911	Cancer, neoplasms and lymphoma	Completed	CD40	Chi Lob 7/4 (a chimeric monoclonal antibody)	Phase 1	Activated B and NK cells [139]
	NCT03922477	Acute myeloid leukemia	Recruiting	CD47	Hu5F9-G4	Phase 1	NA
	NCT00912327	Colorectal cancer	Completed		Imprime PGG	NA	NA
	NCT03248479	Acute myeloid leukemia and myelodysplastic syndromes	Recruiting	CD47	Magrolimab Azacitidine	Phase 1	NA
	NCT01433172	Lung cancer and adenocarcinoma	Completed	CD40 and CCL21	GM.CD40LCCL21 Vaccinations	Phase 1, 2	Well-tolerated
	NCT01904123	Metastatic malignant neoplasm in the brain, metastatic melanoma, recurrent brain neoplasm, recurrent glioblastoma and recurrent malignant glioma	Recruiting	STAT3	STAT3 inhibitor WP1066	Phase 1	NA
	NCT02953509	Lymphoma, non-Hodgkin’s; lymphoma, large B cell, diffuse; and indolent lymphoma	Recruiting	CD47	Hu5F9-G4	Phase 1, 2	NA
	NCT00911560	Neuroblastoma	Recruiting		Adjuvant OPT-821 in a vaccine containing two antigens (GD2L and GD3L) covalently linked to KLH	Phase 1, 2	NA
	NCT01103635	Recurrent melanoma and stage IV melanoma	Completed	CD40	CD40 agonist monoclonal antibody CP-870893, tremelimumab	Phase 1	NA
	NCT03527147	NHL, DLBCL, non-Hodgkin’s lymphoma and diffuse large B cell lymphoma	Recruiting	CD47	Hu5F9-G4	Phase 1	NA
	NCT02157831	Solid tumors	Completed	CD40	CP-870893	Phase 1	NA
	NCT02225002	Advanced solid tumors	Completed	CD40	CP-870893	Phase 1	NA
	NCT00711191	Pancreatic neoplasm	Completed	CD40	CP-870893	Phase 1	Tumoricidal, the depletion of tumor stroma [140]
	NCT00607048	Neoplasms	Completed	CD40	CP-870893	Phase 1	Safe and no long-term [141]
	NCT01456585	Adenocarcinoma pancreas	Completed	CD40	CP-870893	Phase 1	NA
	NCT01103635	Recurrent melanoma and stage IV melanoma	Completed	CD40	CP-870893	Phase 1	NA
	NCT01008527	Melanoma	Completed	CD40	CP-870893	Phase 1	NA
	NCT02482168	Solid tumors	Completed	CD40	APX005M	Phase 1	NA
	NCT01839604	Advanced adult hepatocellular carcinoma and hepatocellular carcinoma metastatic	Completed		AZD9150	Phase 1	Safe and tolerable
	NCT02367196	Hematologic neoplasms	Active, not recruiting	CD47	CC-90002	Phase 1	NA
	NCT02641002	Leukemia, myeloid, acute and myelodysplastic syndromes	Terminated	CD47	CC-90002	Phase 1	NA
	NCT02663518	Hematologic malignancies and solid tumor	Recruiting	SIRPα	TTI-621	Phase 1	NA
	NCT02890368	Solid tumors, mycosis fungoides, melanoma, merkel cell carcinoma, squamous cell carcinoma, breast carcinoma, and papillomavirus-related malignant neoplasm	Terminated	SIRPα	TTI-621	Phase 1	NA
	NCT02665416	Advanced/metastatic solid tumors	Completed	CD40	RO7009789	Phase 1	NA
	NCT02760797	Neoplasms	Completed	CD40	RO7009789	Phase 1	NA
	NCT02304393	Solid tumors	Completed	CD40	RO7009789	Phase 1	NA
	NCT02588443	Pancreatic cancer	Completed	CD40	RO7009789	Phase 1	NA
	NCT03329950	Advanced malignancies	Recruiting	CD40	CDX-301/CDX-1140	Phase 1	NA
	NCT00899574	Breast cancer Breast neoplasms	Completed	TLR7	Imiquimod	Phase 2	NA
	NCT01421017	Breast cancer, metastatic breast cancer and recurrent breast cancer	Completed	TLR7	Imiquimod	Phase 1, 2	Supplemented the response evaluation criteria in solid tumors [142,143]
	NCT04116320	Melanoma, breast cancer, squamous cell cancer, non-small-cell lung cancer, cervical cancer, urothelial carcinoma, ovarian cancer, small-cell lung cancer and esophageal cancer	Recruiting	TLR7	Imiquimod	Phase 1	NA
	NCT03196180	Cervical intraepithelial neoplasia	Active, not recruiting	TLR7	Imiquimod	Phase 1	NA
	NCT00319748	Breast cancer, ovarian cancer, endometrial cancer and cervical cancer	Completed	TLR7	852A	Phase 2	NA
	NCT00719199	Colorectal cancer	Terminated	TLR9	IMO-2055	Phase 1	NA
	NCT01040832	Squamous cell carcinoma of the head and neck	Completed	TLR9	IMO-2055	Phase 2	NA
	NCT01360827	Squamous cell carcinoma of the head and neck	Terminated	TLR9	IMO-2055	Phase 1	NA
	NCT02829723	Advanced solid tumors	Recruiting	CSF1R	BLZ945	Phase 1, 2	NA

## 4. Interaction of Macrophages with NPs

As outlined in Section 3.1, various drugs that target TAMs are currently in preclinical and clinical development (Table 1). However, their application in the clinic has been limited by side effects and the lack of specific targeting properties [144]. To overcome these obstacles, various strategies utilizing NPs loaded with therapeutics and partially functionalized with TAM-targeting motifs have been developed over the last years (Table 2). In this section, we first discuss how macrophages interact with NPs in vitro and in vivo and how their polarization status affects the efficacy of NP uptake. Finally, we discuss the current progress in the development of NPs to deplete or re-educate TAMs for therapeutic purposes.

### 4.1. NP-Based Cancer Therapies Targeting TAMs

Compared to normal tissues, tumor tissues are rich in blood vessels. The rapid growth of tumors and associated blood vessels leads to a decrease in blood vessel wall density and vascular endothelial space and an increase in capillary permeability, resulting in high permeability for macromolecules and NPs, namely by the enhanced permeability and retention (EPR) effect [145]. With the gradual disclosure of the role of TAMs in tumor tissues, it has been found that NPs are significantly enriched in TAM-rich tumor tissues compared to TAM-deficient tissues, and the amount of NP uptake by a single TAM is much larger than that of a tumor cell [146]. Furthermore, TAMs can engulf NPs in large quantities and carry NPs to the anaerobic area of the tumor, thus making a great contribution to the enrichment of drugs at the tumor site [147]. Considering the distribution of TAMs in tumor tissues and their ability to engulf NPs, NP-based strategies targeting TAMs mainly include: (1) the utilization of TAMs as a drug carrier “reservoir” to carry nanomaterials into the central area of solid tumors in order to infiltrate and enrich drugs at the tumor site, (2) activation of TAM autophagy and (3) targeting TAMs with drugs that lead to the transformation of inhibitory (M2 type) macrophages into protumorigenic macrophages (M1 type), thereby inhibiting tumor growth and metastasis [148] (Table 2).

### 4.2. NP Uptake by Macrophages

**Figure 5:**
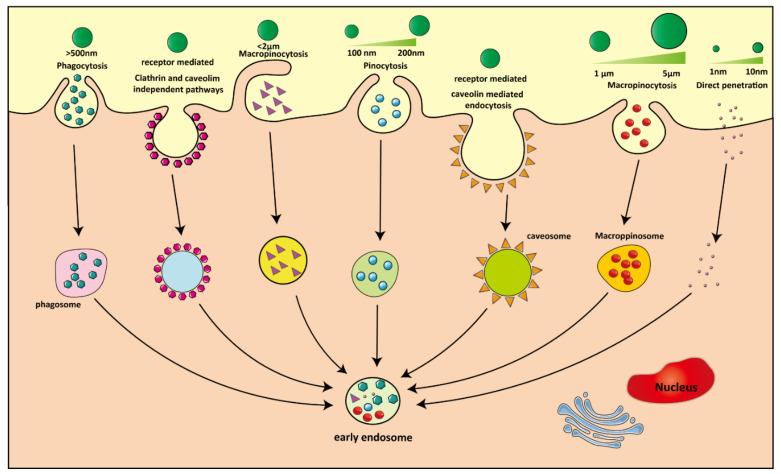
Cellular internalization pathways of nanoparticles (NPs). Depending on their size, NPs enter cells through mechanisms of caveolin- and clathrin-mediated endocytosis, macropinocytosis, phagocytosis or pinocytosis.

Next to neutrophils and dendritic cells, macrophages belong to a group of specialized phagocytes that continuously sample their environment in the process of host defense. The fact that macrophages have multiple subtypes and take on various phenotypes depending on the microenvironment they reside in, can be exploited by macrophage-targeting NPs [149,150]. The surface of NPs can be modified with targeting motifs for specific receptors and/or cell types. Examples of targeting motifs include opsonins derived from serum, such as complement factors or immunoglobins that induce phagocytosis by Fc receptors and complement receptors, respectively, or antibodies, polypeptides and small molecules (i.e., folic acid, hyaluronic acid) [151,152]. Macrophages internalize larger substances (>500 nm) through phagocytosis (Figure 5). In the TME, internalization of particles coated with ligands or motifs (small, repeated, and conserved biological units), such as on the surface of pathogens, occurs via the process of receptor-mediated endocytosis [153]. Receptor-mediated endocytosis is one of the most important processes by which NPs enter cells (Figure 5). This process is mainly mediated by scavenger receptors, integrin receptors and thrombospondin receptors [154,155]. Specific macrophage receptors recognize unique motifs presented on the surface of pathogens, drugs, DNA, or enzymes, and induce a corresponding signaling cascade. In the TME, the surface of M1 macrophages is rich in TLR2/TLR4, CD80, CD86, CD40, CSF1, IL-1R1, INF-γ-R and MHC-II receptors (Figure 3), which are often used as targets for cancer therapy [156,157,158]. For example, the CD40 agonist antibody CP-870893 with tumor targeting function can activate M1 receptor (CD40) during cancer treatment, thereby causing macrophages to rapidly infiltrate tumors and promote tumor matrix depletion in the immunosuppressive tumor microenvironment [140]. M2-type macrophages usually express high levels of Dectin-1, DC-SIGN, mannose receptor (CD206), scavenger receptor A (CD204), IL receptor, MHC-II, CSF-1R, CD163, CCR2, TLR1/8, Arginase 1 (ARG1), galactose receptor and CXCR2 [159].

### 4.3. Effect of NP Physical and Chemical Properties on Cellular Uptake

The extent of cellular uptake of NPs is influenced by several properties, such as NP size, surface charge, hydrophobicity, hydrophilicity, incubation time and NP concentration [160,161], as well as interaction with cell surface receptors and serum proteins [162]. Thus, the desired macrophage-targeting NPs for tumor therapy should have a size that is efficiently taken up by macrophages, be biocompatible, possess a large drug loading capacity and a slow release rate in addition to selectively accumulating at the tumor site.

#### 4.3.1. Size

The size of the NPs directly affects the extent of cellular uptake and intracellular routing (Figure 5). For example, particles with a diameter smaller than 500 nm enter the cells by pinocytosis and particles greater than 500 nm, such as bacteria, dead cells and larger sized NPs, are taken up through phagocytosis [163] (Figure 5). For polymeric NPs (PLA/PLGA), it has been shown that changes from a nanometer to a micrometer range (>1 μm) drastically reduce the cellular uptake rate of NPs [13,164,165]. NPs smaller than 150 nm enter the cell via clathrin-mediated endocytosis [166,167]. In addition, NPs with a diameter less than or equal to the size of cell membrane pores often directly penetrate the cell membrane, such as small metal clusters and dendrimers [168,169], or their uptake is facilitated by cell-penetrating peptides that induce the formation of membrane pores and membrane destabilization [170].

Interestingly, NP size has been shown to be an important determinant in inducing either a Th1 or Th2 response. PLGA NPs of 200–600 nm in size were readily taken up by macrophages and induced a Th1 response (characterized by IFN-γ production and up-regulation of MHC class I molecules), albeit at low antibody titers. In comparison, two–eight-micrometer-sized NPs were tethered to the membrane (were not taken up), but elicited high and long-lasting antibody titers from single-point immunization and showed a Th2 response (characterized by IL-4 secretion, up-regulated MHC class II molecules) [165]. This offers an exciting possibility to modulate an immune response. Depending on the co-administration of adjuvants, PLGA NPs could either induce a M1-type or M2-type response in vivo [171].

Once NPs come into contact with plasma or other protein-containing biological fluids, their surface immediately forms a protein corona with biological activity [172], which affects the interaction of NPs with macrophages [173]. Importantly, the protein corona covering the NP’s surface may hinder the specific interaction between targeting ligands and their receptors. For example, Salvati et al. found that a protein corona shielded a transferrin-functionalized NP’s surface from binding to the transferrin receptor [174]. Contrary, the study by Hoppstädter et al. showed that a protein corona adsorbed on the NP’s surface could be exploited to target M2-macrophage-specific surface receptors, which promoted the uptake of NPs [175]. In line with this finding, SiO2 NPs coated with a hard corona consisting of fibrinogen, immunoglobin G, low-density lipoproteins, high-density lipoproteins or the hormone Kallikrein promoted the binding of NPs to M2 macrophages [176]. As the size and surface area of NPs increases, the unspecific adsorption capacity of proteins from body fluids by the NP surface increases and plays a more important role in NP uptake and interaction with cell membrane receptors [177].

In vivo, the size of the NPs determines whether the NPs enter the lymphatic capillaries or remain at the injection site. For example, preclinical studies conducted in mice found that NPs with diameters between 10 and 100 nm quickly reached the kidneys through capillaries and were easily secreted, while NPs with diameters greater than 130 nm could not pass through the glomerular basement membrane [178,179]. Therefore, to avoid clearance by the mononuclear phagocytic system before reaching the TME, and to meet the needs of drug loading, NPs with a diameter in the size range of 100–200 nm are preferred [30]. In addition, NPs with a diameter in the range of 100–200 nm can leak through the tumor’s blood vessels (EPR effect) and are quickly adsorbed by dendritic cells in the lymph nodes [180,181]. Similar to the EPR effect in solid tumors, the vascular permeability of inflamed joints in rheumatoid arthritis allows for passive targeting of NPs to the inflammation side. The targeting efficacy of PEGylated liposomes of different sizes (70, 100, 200 and 350 nm) and surface charges were evaluated in vivo in a mouse model by near-infrared fluorescence imaging [182]. The results showed that liposomes with 100 nm diameter and a slight negative charge had better in vivo circulation times and inflamed joint targeting than other liposomes did [182].

#### 4.3.2. Shape

It has been demonstrated that the shape (spherical, oblate, cubic, worm-shaped, rod-shaped) of the NPs plays a vital role in their interaction with the cell membrane [183,184,185]. Li et al. showed that the shape of NPs affects the immune response of macrophages and the efficacy of passive and targeted NP uptake by macrophages [186]. Spherical glyco-NPs were internalized by clathrin- and caveolin-mediated endocytosis, while cylindrical glyco-NPs were mainly dependent on clathrin-mediated endocytosis. Interestingly, the authors found that longer and cylindrical NPs preferentially induced an IL-6-dependent inflammatory response, compared to shorter cylindrical NPs. Similar, *Niikura* et al. reported that the size and shape of AuNPs coated with West Nile virus membrane protein for vaccine purposes determined the type of inflammatory response [187]. Rod-shaped AuNPs induced significant levels of inflammasome-dependent cytokine secretion (IL-1β, IL-18), while spherical AuNPs significantly induced the inflammatory cytokines TNF-α, IL-6, IL-12 and GM-CSF.

A comparative uptake study in human leukocyte populations found that macrophages take up small rod-shaped AuNPs (15 and 50 nm) with greater ease compared to spherical AuNPs [188]. A possible explanation for why differently shaped NPs induced different cellular responses was provided by Hinde et al. [189]. The authors found that polymeric NPs with different shapes (but identical surface charges) moved across various cellular barriers (plasma membrane, endosomal and lysosomal membranes as well as nuclear envelopes) [189]. However, rods and worms, but not micelles and vesicles, could diffuse to the nucleus via passive diffusion, thus improving their nuclear access rate [189]. In summary, NP shape is an important point to consider when designing NP systems to deliver drugs across cell membranes to macrophages.

#### 4.3.3. Surface Charge

The surface charge of NPs plays a key role in determining the interaction with the cell membrane, the mechanism of NP uptake, intracellular localization, immune response and cytotoxicity. For example, the study by Du et al. showed that a positive surface charge promoted NP uptake by Caco-2 cells (human colon adenocarcinoma cell line) and small intestinal epithelial cells in vitro and in vivo, respectively [190]. There are many molecules with different charges in biological systems. In vitro studies have found that phosphatidylserine phospholipids, the major components of the plasma membrane, carry anions that cause the cell membrane to be negatively charged [191]. Therefore, cationic NPs interact with the cell membrane through electrostatic interaction. In vitro studies using RAW 264.7 murine macrophages found that both positively and negatively charged NPs showed higher uptake rates after opsonization in fresh mouse serum, but positively charged NPs induced higher cytotoxicity rates [192]. In vivo biodistribution studies demonstrated that undesirable liver uptake was increased for highly positively or negatively charged NPs, likely due to active phagocytosis by macrophages (Kupffer cells) in the liver. However, liver uptake was low and tumor uptake was high when the surface charge of NPs was slightly negative. This study suggests that a slight negative charge may be introduced to the NP surface to reduce the undesirable clearance by the reticuloendothelial system (RES) and to increase tumor localization [192]. Similar results have been obtained for chitosan NPs, demonstrating that NPs with slight negative charges and a size of 150 nm accumulated in tumors more efficiently [193].

Getts et al. showed that cationic NPs can induce TAM proinflammatory responses; anionic NPs are significantly less immunogenic than cationic NPs [194]. NPs are usually coated with polymers (such as PEG) or zwitterionic structures to improve their biocompatibility, increase their stability and extend their circulation time in the body [24,195,196].

#### 4.3.4. Surface Hydrophobicity

NPs hydrophobicity directly affects the interaction with cells in vivo. Hydrophobic NPs are easily covered by protein complement forming a corona, which further enhances the surface hydrophobicity of NPs [197]. For example, Qianhui et al. found that hydrophobic NPs easier adsorb proteins, while hydrophilic NPs have a higher protein exchange rate [197]. Coating NPs with PEG and its analogs can not only prevent the formation of a protein corona [197], but also enhances the NPs hydrophilicity and increases the possibility of reaching the tumor site.

### 4.4. Effect of Macrophage Polarization Status on NP Uptake

The literature suggests that the polarization status of macrophages severely affects NP uptake. Jones et al. showed that mouse strains that are prone to Th1 immune responses clear 300 nm cylindrical PEG hydrogel NPs at a slower rate than Th2-prone mice. The NP uptake capacity was increased when macrophages from Th1 strains were differentiated towards M2 macrophages, while M1 macrophages were more likely to take up NPs larger than 500 nm in diameter [198]. Thus, the polarization status of macrophages influences NP clearance capacity. Hoppstadter et al. investigated the uptake of fluorescent silica NPs in different types of macrophages. The results showed that M2 macrophages obtained from lung tumors absorbed more NPs than M1 macrophages isolated from the surrounding lung tissue [175]. MacParland et al. measured the uptake of 100-micrometer-sized AuNPs by different macrophage subtypes, and the results showed that M2c macrophages had the strongest affinity for ingesting AuNPs, followed by M2 macrophages and M1 macrophages, while monocytes had the weakest affinity for NPs [199]. The uptake pattern of silica NPs by M2c macrophages and M1 macrophages in vitro was similar to that of AuNPs [200,201]. Thus, these studies suggest that M2 macrophages promote the internalization of NPs [200].

In contrast to these findings, Herd et al. found a higher uptake of Stöber silica NPs of 130 nm in size in M1 macrophages compared to M2 macrophages [200]. Importantly, they also found NPs to preferentially accumulate in liver and spleen tissues in vivo in macrophages with a Th1/M1 phenotype response. An explanation for the preferential uptake of silica NPs by M1 macrophages is that some M1 surface receptors have been implicated in promiscuous poly anionic and silanol binding in vivo [200]. In addition, a study by Pajarinen et al. found that the uptake of titanium NPs by M1 macrophages in vitro was stronger than that of M2 macrophages [202].

In summary, while the polarization status of macrophages clearly determines the efficacy of NP uptake, controversy remains on which macrophage subset takes up more NPs in vitro and in vivo. This controversy might be explained by differences in physicochemical properties of the NPs (size, shape and material) tested in the studies, which can lead to preferential uptake behavior in M1 or M2 subtypes.

### 4.5. Modulation of TAMs by NPs via Passive Targeting In Vivo

Table 2 summarizes various strategies for targeting TAMs in the course of cancer immunotherapy by different types of NPs. In vivo, NPs need to overcome many physiological barriers, including clearance by immune cells in the liver and spleen, various endothelial membranes, interstitial spaces, the plasma membrane, endosomal/lysosomal membranes and finally the nuclear membrane and pore complex [203]. Upon intravenous injection, a fraction of NPs reaches the TME primarily via the EPR effect, while another part is taken up by the macrophages of the liver and spleen, which are in direct contact with the bloodstream [204,205]. In addition, the abnormal structure of blood vessels in tumors allows the penetration of NPs, while defects in the lymphatic system facilitate the retention of NPs in the TME, where they slowly release their payload, such as antitumor drugs [206,207]. Several TAM-targeting strategies exploit the EPR effect and/or the intrinsic phagocytotic capacity of TAMs, thus circumventing the need for specific targeting moieties attached to the surface of NPs. In this section, we discuss NP-based passive targeting strategies to TAMs that do not utilize specific targeting motifs.

Liposomes have been exploited in many studies as delivery system for drugs that deplete or modulate TAMs or inhibit their recruitment. For example, Li et al. demonstrated that liposome loaded with C6-ceramide can mitigate the immunotolerant TME of liver cancer by decreasing the frequency of TAMs and the ability of TAMs to suppress the antitumor immune response by modulating ROS signaling and increasing the M1 phenotype [31]. NPs can protect siRNA molecules from rapid degradation in the blood stream. Leuschner et al. used CCR2-siRNA-loaded liposomes of 70–80 nm in size to silence CCR2 mRNA expression, which is required for monocyte recruitment to tumors [208]. After intravenous injection, the liposomes showed a half-life of 8.1 min and accumulated in the spleen and bone marrow, where they delivered siRNA into monocytes expressing high levels of Ly6C, which are precursors of TAMs. This liposome formulation potently decreased the number of TAMs and inhibited tumor growth [208]. Similar, Shen et al. developed monocyte-targeted, siCCR2-loaded, cationic lipid-assisted NPs and reduced the recruitment of monocytes to tumor tissues [209]. As discussed in Section 3.1, STAT3 inhibitors have been shown to promote the M1 phenotype of TAMs. Jose et al. manipulated liposomes to co-deliver curcumin and STAT3 siRNA to treat melanoma. The curcumin/siRNA-loaded liposomes inhibited tumor growth compared to either liposome-curcumin or STAT3 siRNA alone. The antitumor activity of curcumin/siRNA-loaded liposomes was associated with suppressed STAT3 activity and the repolarization of M1 macrophages towards the M1 type [210]. Other drugs, whose therapeutic effect against TAMs has benefited from NPs, are clodronate and bisphosphonates [211]. TAMs play a pivotal role in tumor growth by promoting angiogenesis, and clodronate encapsulated in liposomes (clodrolip) efficiently depleted these phagocytic cells in the murine F9 teratocarcinoma and human A673 rhabdomyosarcoma mouse tumor models, resulting in the significant inhibition of tumor growth [212]. Similar, clodronate encapsulated in liposomes administered into a mouse model of melanoma depleted TAMs and inhibited the monocyte chemoattractant protein 1 (MCP-1) pharmacologically, thereby reducing macrophage recruitment [213]. The bisphosphonate zoledronate has previously been shown to promote the M1 type in macrophages cultured in vitro in a medium conditioned with soluble factors of breast cancer cells [214]. When encapsulated into liposomes, it enhanced levels of the proinflammatory signals iNOS and TNF-α. Rajan et al. showed that PEGylated liposomes encapsulating alendronate efficiently targeted macrophages in the immunocompetent TC-1 murine tumor model, reduced tumor growth and increased disease-free survival [215]. VEGF is a major inducer of tumor angiogenesis and is also required for macrophage recruitment (Table 2). Clodronate encapsulated in liposomes for cancer therapy, in combination with angiogenesis inhibitors, such as anti-VEGF antibodies, has been shown to be more efficient than single-modality treatments, targeting the recruitment hematopoietic precursor cells that stimulate tumor growth [212].

In addition, VEGF and placental growth factor (PIGF), which are overexpressed in M2 TAMs and breast cancer cells, have been shown to work synergistically in mediating tumor progression and immunosuppression. Song et al. developed combinational antitumor immunotherapy using PEG and mannose doubly modified trimethyl chitosan NPs to deliver VEGF and PIGF siRNA to both M2 TAMs and breast cancer cells for antitumor immunotherapy [127]. These pH-sensitive NPs showed prolonged blood circulation times and efficiently accumulated in M2 TAMs and breast cancer cells via mannose-mediated active targeting and passive targeting, respectively. The co-delivery of siVEGF and siPIGF reduced the proliferation of tumor cells and reversed the TME from pro-oncogenic to antitumoral and suppressed breast tumor growth and lung metastasis.

In another approach, PEGylated liposomes functionalized with anti-CD40 and CpG oligonucleotides were developed. CpG oligonucleotides are ligands for Toll-like receptors (TLR) and potent immunostimulatory agents, whereas CD40 ligation triggers a signaling mechanism to promote an antitumor T cell response [216]. Anti-CD40/CpG-liposomes successfully sequestered anti-CD40 and CpG in vivo, significantly inhibited tumor growth and induced a survival benefit in a B16F10 murine model of melanoma (Table 2).

Polymeric NPs have been extensively used to target TAMs. Jung et al. formulated epoxide-modified lipid–polymer hybrid NPs termed 7C1 containing CX3CL1 siRNA to block monocyte recruitment [217]. Their results showed that 7C1-CX3CL1 siRNA significantly reduced the CX3CL1 level in vivo and consequently also reduced Ly6C^lo^ monocyte recruitment. This study revealed an immunosuppressive function of Ly6C^lo^ monocytes (Table 2). Recently, Cavalcante et al. demonstrated that naked or hyaluronic (HA)-coated PeiPLGA NPs encapsulating methotrexate injected intratumorally modified the tumorigenic course of 4T1 breast cancer by modulating the TME, leading to a reduction in primary tumor size and metastases. Interestingly, the application of HA-PeiPLGA NPs promoted the repolarization of M2 macrophages towards the M1 type, reduced the levels of IL-10 and down-regulated the levels of STAT3 and NF-κB [117]. In addition, HA-PeiPLGA NPs reduced the levels of CCL22, IL-10 and TGF-β expression, as well as the recruitment of Tregs and enhanced the cytotoxicity of CD8 T cells in the TME. Another strategy aimed at modifying TAMs in the TME into tumor-suppressive macrophages by local delivery of IL-12. To this end, Wang et al. synthesized microenvironment-responsive poly β-amino ester-based NPs with IL-12 as a payload [218]. The NPs exhibited enhanced tumor accumulation, and significantly extended the circulation half-life and therapeutic efficacy of encapsulated IL-12 compared to free IL-12.

Polymeric chitosan NPs have also been used as an intranasal delivery system for siRNAs targeting galectin-1 expression for the treatment of glioblastoma multiforme (GBM). Galectin-1 is overexpressed in GBM and drives chemo- and immunotherapy resistance. Interestingly, when delivered intranasally and after reaching the TME via nose-to-brain transport, galectin-1 siRNA chitosan NPs silenced galectin-1 in the TME and induced a remarkable switch in the TME composition, including a reduction in macrophage polarization from M1 towards the M2 phenotype during GBM progression [219].

As discussed in Section 3.1, TLR signaling can modulate macrophage polarization. Rodell et al. identified that R848, a TLR7/TLR8 agonist, efficiently induces the M1 phenotype in vitro. Based on this finding, they developed R848-loaded cyclodextrin NPs [220]. After in vivo administration, the NPs accumulated in macrophage-rich tissues over time and demonstrated high polarization efficiency in multiple tumor models. Moreover, R848-loaded cyclodextrin NPs protected mice in tumor rechallenge experiments. Furthermore, the combination of R848-loaded cyclodextrin with an anti-PD-1 checkpoint blockade greatly improved immunotherapy response rates.

Next to liposomes and polymeric NPs, inorganic NPs, which are characterized by their small sizes, large surface areas and various surface modifications, have also attracted attention for TAM-based cancer immunotherapy. For example, Zhao et al. showed that poly(I:C)-loaded amino-modified ferumoxytol NPs could effectively enhance the antitumor immune response, thereby improving cancer immunotherapy and promoting the regression of primary and metastatic melanoma [221]. In vitro studies further showed that ferumoxytol NPs activated the NF-κB signal transduction pathway in macrophages and promoted the M1 phenotype, which prevented tumor cell growth [221].

Multi-walled carbon nanotubes (MWCNTs) belong to the family of nanovectors and have been evaluated as a potential delivery system for brain tumor therapy in the GL261 murine intracranial glioma model. After intratumoral injection, nearly 10–20% of total cells demonstrated MWCNT internalization. Importantly, most MWCNT uptake occurred by TAMs, suggesting that MWCNTs could potentially be used as a non-toxic vehicle for targeting TAMs in brain tumors [222]. Mesoporous silica nanoparticles (MSNs) have been extensively studied for systemic drug delivery. Kwon et al. developed MSNs of 200 nm in size with a larger pore sizer than conventional MSNs, allowing the encapsulation of larger therapeutic biomolecules [223]. XL-MSNs were used to deliver IL-4 in vivo to macrophages and to polarize them towards the anti-inflammatory M2 type (Table 2). Upon intravenous injection, XL-MSNs targeted phagocytic myeloid cells, including macrophages, and successfully triggered M2 macrophage polarization in vivo, demonstrating the clinical potential of XL-MSNs for the modulation of the immune system via targeted delivery of cytokines.

Inhibition of CSF1R signaling has been shown to suppress macrophage infiltration and to reduce tumor growth [224]. Ramesh et al. developed dual-kinase inhibitor-loaded supramolecular nanoparticles (DSNs) for the concurrent and sustained inhibition of the CSF1R and MAPK signaling pathways [225]. When tested in the aggressive 4T1 breast cancer model, the DSNs accumulated in TAMs at a significantly higher concentration, increased M1-like phenotype at a significantly higher proportion and improved antitumor efficacy as compared to a combination of single-inhibitor NPs or small-molecule inhibitors.

Several NPs harbor intrinsic properties that regulate the balance between M1 and M2 macrophages in vitro and in vivo by modulating TAM-related signaling pathways. For example, surface functionalization (-COOH and -NH2) of polystyrene NPs has been shown to impair the expression of the scavenger receptor CD163 and the inhibitor cell surface transmembrane glycoprotein CD200 receptor (CD200R) on M2 macrophages, as well as the release of IL-10, without affecting the expression of M1 markers [226]. Feito et al. used graphene oxide nanosheets (FITC-PEG-graphene oxide) modified with PEG amine functional groups and a fluorescein isothiocyanate label to explore their effect on the polarization of murine peritoneal macrophages [201]. They found an increase in M2 macrophage phenotype after nanosheet treatment, likely promoting the percentage of M2 macrophages for tissue repair [201]. Interestingly, while Au NPs were reported to have intrinsic properties that promote the M2-like phenotype in RAW 264.7 cells in vitro, CaCO3-encapsulated Au NPs induced the expression of the M1 biomarker and inflammatory cytokines [227]. In another study, hydroxyl dendrimers were combined with the CSF-1R inhibitor BLZ945 (D-BLZ) to target TAMs in brain glioblastoma. The authors showed that the conjugation of BLZ945 to dendrimers enabled sustained release in intracellular and intratumor conditions. A single systemic dose of D-BLZ targeted to TAMs decreased the protumorigenic phenotype of TAMs and promoted the infiltration of cytotoxic T cells, resulting in prolonged survival and an ameliorated disease burden compared to free BLZ945 [224]. In summary, targeting NPs to TAMs does not necessarily require the presence of targeting motifs on the surface of NPs. Due to several factors, such as the EPR effect, the positioning of TAMs in the TME, as well as their phagocytotic nature, passive targeting strategies have been shown to lead to the accumulation of NPs in TAMs in vivo. However, these strategies are not TAM-specific and lead to the global uptake of NPs in TME cellular subsets, including immune cells. Thus, whether passive targeting is suitable for the delivery of therapeutics to TAMs must be carefully evaluated.

### 4.6. Targeting NPs to TAMs via Specific Surface Receptors

The elimination or modification of TAMs by targeting NPs to TAM surface receptors is regarded as a promising method in cancer immunotherapy. To date, NPs encapsulating DNA, vaccines, oligonucleotides and imaging agents to TAMs have been targeted to macrophages via membrane receptors (Table 2). Mannose receptors, scavenger receptors (CD163) and CD200R are highly expressed on the surface of M2 macrophages, and thus represent potent targets for NP delivery to TAMs [47].

#### 4.6.1. CD163

For example, antibody-decorated liposomes loaded with doxorubicin could be specifically targeted to TAMs in vivo after injection into mice via the hemoglobin scavenger receptor CD163 [228]. Specific depletion of CD163+ TAMs resulted in a massive infiltration of activated T cells, the mobilization of inflammatory monocytes and tumor regression.

#### 4.6.2. Mannose Receptor

In addition, carbohydrates, such as mannose, galactose and glucomannan, can also be employed as targeting motifs. Mannose has been shown to continuously promote the internalization of mannosylated molecules [100]. Therefore, decorating NPs with mannose to target the mannose receptor for the selective delivery of therapeutics is a common method in the field of drug delivery. Experiments have shown that primary macrophages took up 4-fold more siRNA when pH-responsive polymeric micelles encapsulating siRNA were mannosylated compared to naked NPs [229]. Several polymeric NPs, including PLGA, have been decorated with mannose to target the mannose receptor on TAMs. Shi et al. formulated mannose-decorated PLGA NPs to co-deliver ICG, ammoniumhydrogencarbonate (NH_4_HCO_3_) and titanium dioxide (TiO_2_) to tumors [230]. Upon an external laser trigger, the NPs promoted the formation of reactive oxygen species, which led to the reprogramming of TAMs towards the antitumor M1 phenotype in vitro and in vivo. Zhu et al. decorated acid-sensitive PEG-modified PLGA NPs with mannose and examined their uptake by TAMs in vivo [231]. The authors demonstrated that the NPs efficiently targeted TAMs via mannose–mannose receptor interactions after acid-sensitive PEG shedding in the acidic TME, while their uptake by normal macrophages in the spleen and liver was significantly reduced due to effective PEG shielding at neutral pH. In another example, researchers designed cationic nanohydrogel NPs containing α-mannosyl functional groups and CSF-1R siRNA to specifically target TAMs through the mannose receptor [232]. SiRNA-loaded mannosylated NPs preferentially targeted mannose receptors on M2 macrophages and effectively reduced expression of CSF-1R. The targeted immunomodulatory response only occurred in M2 macrophages and did not affect the expression state in M1 macrophages [232]. Research by Niu et al. showed that when sufficient TAMs are present in the tumor, PEGylated, mannose-modified PLGA-DOX NPs could effectively deliver DOX to the tumor site, thereby inhibiting tumor growth and reducing the number of TAMs in TME [233]. In another example, hybrid NPs made of Pluronic^®^ -F127 polymer and tannic acid with varying mannose densities showed superior uptake behavior in M2-polarized U937 macrophages in vitro [234]. Metformin (Met), a popular drug used to treat diabetes, also exhibits anticancer activity. Recently, Met has been found to efficiently repolarize M2-like TAMs towards the M1 phenotype to inhibit tumor growth and metastasis through the AMPK-NF-κB signaling pathway [235]. Wei et al. developed mannose-modified macrophage-derived microparticles (Man MPs) encapsulating metformin [236]. Met@Man-MPs efficiently targeted M2-like TAMs and repolarized them into the M1-like phenotype. In addition, Met@Man-MPs increased the recruitment of CD8+ T cells to the TME and decreased the immunosuppressive infiltration of myeloid-derived suppressor cells and Tregs.

Wang et al. developed bioresponsive polymeric NPs (P3AB) that could selectively eliminate TAMs. The NPs consisted of PEG, PLGA and a MMP-cleavable peptide that formed the shell, while a bisphosphonate–glucomannan conjugate with affinity for macrophage mannose receptors formed the core. While in this strategy the NPs were not targeted specifically to TAMs, this strategy utilized MMPs overexpressed in TAMs to cleave the NP shell to expose the bisphosphonate–glucomannan conjugates in order to eliminate TAMs. Systemically administered P3AB polymeric complexes were taken up by TAMs and led to a potent reduction in TAM viability [237], effectively inhibiting tumor growth and restoring local immunosurveillance in vivo.

#### 4.6.3. C-Type Lectin

TAMs express high levels of macrophage galactose-specific C-type lectin (MGL) [238]. Decorating NPs with galactose moieties can facilitate the recognition and uptake of NPs by TAMs. Han et al. developed baicalin (a flavone glycoside)-loaded PLGA NPs containing an antigenic peptide (Hgp 10025–33, Hgp) and a Toll-like receptor 9 agonist (CpG), coated with a galactose-inserted erythrocyte membrane, which could actively target TAMs and showed enhanced cell uptake in vitro and targeting in vivo [239] (Table 2). In addition, the biomimetic NPs containing baicalin, Hgp and CpG, or baicalin- and galactose-inserted erythrocyte membranes significantly repolarized the TAMs from the M2-like type towards the M1-like type, both in vitro and in vivo, which led to a significant enhancement of the antitumor T cell response. Another approach focused on repolarizing TAMs to antitumor M1 macrophages by administration of redox/pH dual-responsive nanovectors encapsulating with PEG-PLL (sPEG) copolymers/galactose-functionalized n-butylamine-poly(l-lysine)-b-poly(l-cysteine) polypeptides (GLC) for delivery of miR155. miR155 had previously been shown to significantly attenuate the cytokine production in TAMs via targeting C/EBPβ [240] and repolarized protumoral [240] and re-polarized pro-tumoral M2 TAMs into antitumor M1 macrophages in vitro [241]. The results showed that administration of sPEG/GLC/155 nanocomplexes increased miR155 expression in TAMs about 100–400-fold both in vitro and in vivo, and efficiently repolarized immunosuppressive TAMs towards antitumor M1 macrophages by elevating M1 macrophage markers (IL-12, iNOS and MHC II) and suppressing M2 macrophage markers (Msr2 and Arg1) in TAMs [242]. Huang et al. developed PEG-histidine-modified alginate (PHA) NPs encapsulating galactosylated cationic dextran with nucleic acids. Using this system, the authors combined CpG oligonucleotides, anti-IL-10 and anti-IL-10 receptor oligonucleotides [243]. After intravenous injection in an allograft hepatoma murine model, the acidic environment of the TME triggered an alteration in the charge of PHA from negative to positive, which led to the dissociation of PHA from the complex and exposed the galactosylated cationic dextran–ODN complex, which led to the predominant uptake of NPs in TAMs via MGL. As a consequence, the authors observed suppression of protumor functions and stimulation of the antitumor activities of TAMs [243].

Moreover, self-assembled glyco-NPs, composed of hydrophobic block polystyrene (PS) and a hydrophilic glycoblock have been shown to successfully induce the polarization of mouse primary peritoneal macrophages from M2 to the inflammatory type M1 in vitro and in vivo [244]. The glyco-NPs were coated with three sugars (d-galactopyranoside, α-mannopyranoside and l-fucopyranoside), which served as ligands for C-type lectin receptors on macrophages. To increase the selectivity of M2 TAM targeting, Cieslewicz et al. screened a peptide phage library and identified M2pep as a promising alternative to the traditional ligands [245]. When conjugated to a proapoptotic peptide, it could induce toxic effects in M2 macrophages. Pang et al. modified the surface of PLGA NPs with M2pep and encapsulated the CSF-1/CSF-1R pathway inhibitor, PLX3397 [246]. The results showed that M2pep conjugation increased the uptake of PLGA NPs by M2-polarized macrophages, improved the antitumor efficacy of PLX3397 and helped to attenuate tumor growth in the mouse B16F10 melanoma model.

#### 4.6.4. Scavenger Receptor B Type 1

In another approach, Qian et al. developed lipid NPs specifically targeting M2-like TAMs by including the α-peptide (a scavenger receptor B type 1 (SR-B1)-targeting peptide) linked to M2pep [126]. The lipid NPs were loaded with CSF-1R-siRNA to specifically block the survival signal of M2-like TAMs and to deplete them from melanoma tumors. The results showed a dramatic elimination of M2-like TAMs, decreased tumor size and prolonged survival. In addition, this targeting strategy overall reduced the immunosuppressive state of the TME by reducing expression of IL-10 and TGF-β, while increasing the immunostimulatory cytokines IL-12 and IFN-γ. Furthermore, siRNA-carrying lipid NPs increased CD8+ T cell infiltration (2.9-fold) and down-regulated expression of the exhaustion markers PD-1 and Tim-3.

#### 4.6.5. Sialic Acid Binding Receptors

Sialic acid binding receptors (siglecs) are overexpressed on the surface of TAMs and thus can be utilized for TAM-specific targeting by decorating NPs with sialic acid. For example, epirubicin-loaded liposomes were decorated with sialic acid–cholesterol conjugates to improve the delivery of epirubicin to TAMs [247].

#### 4.6.6. Legumain

In an alternative approach, hydrazinocurcumin-encapsulated liposomes, a synthetic analog of curcumin with improved water solubility, cell permeability and bioavailability, were used to target legumain, a protease specifically expressed on TAMs. Hydrazinocurcumin was used to inhibit STAT3 activity and to re-educate TAMs. Furthermore, hydrazinocurcumin down-regulated a series of immunosuppression-related proteins and downstream target molecules of STAT3, thereby limiting the proliferation and migration of 4T1 breast cancer cells in vitro, as well as tumor growth, angiogenesis and metastasis in vivo [248].

Instead of focusing on specific receptors, cell-membrane-coated NPs have emerged as a promising antitumor therapeutic strategy, due to their enhanced blood circulation, immune compatibility and tissue-targeting capacities [249]. Recently, this strategy has been expanded to TAMs. TAM membrane (TAMM) derived from 4T1 primary breast tumors was isolated and coated onto the surface of upconversion NPs (UCNPs) made of rare-earth metals (namely, NaYF4:Yb, Er@NaYF4). The TAMM was rich in CSF1-R, which could be exploited to capture the CSF1 secreted by tumor cells in the TME upon injection of UCNPs into tumor-bearing mice. This blocked the interaction between TAMs and cancer cells and the activation of downstream signaling pathways responsible for the polarization of TAMs to the immunosuppressive phenotype. In particular, after coating the TAMM-UCNPs with photosensitizer and applying photodynamic therapy, macrophages in the TME could be polarized from an immunosuppressive M2-like phenotype to a more inflammatory M1-like state, while the PDT effect induced immunogenic cell death. Overall, this dual approach enhanced the antitumor immune response by the activation of antigen-presenting cells and production of tumor-specific effector T cells in metastatic tumors. Natural NPs, such as exosomes, have been shown to transfer cargo between neighboring cells of the tumor microenvironment, and have been implicated in promoting tumor progression. Several non-coding RNA molecules, such as microRNAs, were shown to be present in these vesicles [250]. Trivedi et al. manipulated the miRNA content of secreted exosomes by the genetic transfection of tumor cells using dual-targeted hyaluronic-acid-based nanoparticles encapsulating plasmid DNA encoding for wild-type p53 (wt p53) and microRNA-125b [251]. These altered miRNA levels in the exosomes mediated macrophage repolarization towards a more proinflammatory/antitumor M1 phenotype.

**Table 2:** Overview of NP-based TAM-targeting strategies in cancer therapy.

Category	NP Materials	Payload	Targeted Receptor	TAM-Targeting Strategy	Ref.
Polymeric NPs	Hyaluronic-acid-coated Pei-PLGA NPs	Methotrexate (MTX)	CD44	Reprogramming TAMs	[117]
	Chitosan NPs	Gal-1 siRNA	Passive targeting	Modulation of TME	[219]
	PEG-sheddable, mannose-modified PLGA NPs		Mannose receptor	Targeting TAMs	[231]
	Cationic polymeric NPs	siCCR2	Passive targeting	Inhibition of monocyte recruitment	[209]
	Carboxyl- and amino-functionalized polystyrene NPs		Passive targeting	Reprogramming TAMs	[226]
	PEGylated silk fibroin NPs	Silk and fibroin	Passive targeting	Reprogramming of TAMs	[207,251]
	Hyaluronic acid–PEG blend NPs	p53 plasmid	Passive targeting	Reprogramming of TAMs	[251]
	MMP-responsive PLGA NPs	Bisphosphonate-glucomannan	Mannose receptor	Interference with TAMs survival	[237]
	Mannose-modified polymeric micelles	siRNA	Mannose receptor	Depletion of TAMs	[229]
	PLGA NPs	Radachlorin	Passive targeting	Depletion of TAMs	[252]
	Mannose-modified polymeric NPs	siRNAs to modulate NF-κB signaling	Mannose receptor	Reprogramming of TAMs	[253]
	PEG and mannose doubly modified trimethyl chitosan NPs	VEGF siRNA/PIGF siRNA	Mannose receptor and passive targeting	Reprogramming TAMs	[127]
	Nanovectors made of galactose-functionalized n-butylamine-poly(l-lysine)-b-poly(l-cysteine) polypeptides coated with DCA-grafted sheddable PEG-PLL copolymers	miR155	C-type lectin (MGL) receptor	Reprogramming TAMs	[242]
	Mannose/acid-sensitive PEG-modified PLGA NPs	Doxorubicin (DOX)	Mannose receptor	Depletion of TAMs	[233]
	M2pep-coated PLGA NPs	PLX3397	Passive targeting	Depletion of TAMs	[246]
	Biomimetic NPs	CpG, baicalin and antigenic peptide	Passive targeting	Reprogramming TAMs	[239]
	Microenvironment-responsive polymeric (P1) NPs	IL-12	Passive targeting	Reprogramming TAMs	[218]
	Mannan-coated PLGA NPs	Didanosine	Mannose receptor	Depletion of TAMs	[206]
	Mannose-modified PLGA NPs	ICG, NH4HCO3 and titanium dioxide	Mannose receptor	Reprogramming of TAMs	[230]
	miR155-loaded sPEG/GLC (sPEG/GLC/155) NPs	miR155	Passive targeting	Reprogramming TAMs	[242]
	Arginine NPs	Cas9 and gRNA	Passive targeting	Increased phagocytosis of tumor cells by macrophages	[254,255]
	PEG-histidine-modified alginate NPs	Oligodeoxynucleotide and galactosylated cationic dextran	Macrophage galactose-type lectin (Mgl)	Reprogramming TAMs	[243]
	Cyclodextrin NPs	R848	Passive targeting	Reprogramming of TAMs	[220]
Lipid-based NPs	Liposomes	C6-ceramide (LipC6)	Passive targeting	Depletion and reprogramming of TAMs	[31]
	MMP2-sensitive apoptotic body-mimicking NPs and phosphatidylserine-modified NPs	Phosphatidylserine	MMP2-sensitive	Depletion of TAMs	[256]
	Cationic liposomes	Curcumin and anti-STAT3 siRNA	Passive targeting	Reprogramming of TAMs	[210]
	Sialic acid–cholesterol conjugate-modified liposomes	Epirubicin and sialic acid	Siglec receptors	Depletion of TAMs	[247]
	Liposomes	CCR2 siRNA	Passive targeting	Depletion of TAMs	[208]
	Liposomes	Hydrazinocurcumin	Legumain	Reprogramming of TAMs	[248]
	Clodronate liposomes	Clodrolip	Passive targeting	Depletion of TAMs	[212,213]
	Liposomes	Zoledronate	Passive targeting	Depletion of TAMs	[214]
	PEGylated liposomes	Alendronate	Passive targeting	Reprogramming of TAMs	[215]
	Long-circulating liposomes	Simvastatin	Passive targeting	Interference with TAM survival	[257]
	PEGylated liposomes	Anti-CD40 and CpG oligonucleotides	CD40	Reprogramming of TAMs	[216]
	Supramolecular lipid NPs	Dual-kinase (MEK and CSF1R) inhibitor	Passive targeting	Reprogramming of TAMs	[225]
	Sorafenib-loaded lipid NPs (SLNPs)	Sorafenib	Passive targeting	Relieve of the immunosuppressive TME	[258]
	α-peptide- and M2pep-linked lipid NPs	Anti-CSF-1R siRNA	Scavenger receptor B type 1 (SR-B1) and M2 macrophage binding peptide	Depletion of TAMs	[126]
	Clodronate-loaded liposomes	Bindarit and clodronate	Passive targeting	Inhibition of macrophage recruitment	[213]
Inorganic NPs	Mesoporous silica NPs	IL-4	Passive targeting	Reprogramming of TAMs	[223]
	Fe2O3 NP	TLR3 agonist, poly (I:C)	Passive targeting	Reprogramming of TAMs	[221]
	Fe3O4/PLGA NPs	Anti-CD206	CD206	Reprogramming of TAM	[149]
	Ca-CO_3_/Au-NPs		Passive targeting	Reprogramming of TAMs	[227]
	Au@SiO2 NPs (GNPs)	Anti-CD163	CD163	Depletion of TAMs	[259]
	Stöber silica NPs		Passive targeting	Sequestration in M1 TAMs	[200]
	Peptide/hyaluronic acid/protamine/CaCO_3_/DNA-NPs	pDNA and IL-12	CD44	Reprogramming of TAMs	[260]
	Hyaluronic acid-manganese dioxide (MnO2) NPs	Doxorubicin	CD44	Reprogramming of TAMs	[261]
	αvβ3-integrin-targeting (αvβ3-antagonist, a quinalone nonpeptide)-perfluorocarbon NPs	MYC inhibitor	Vitronectin receptor αvβ3	Depletion and reprogramming of TAMs	[262]
	MnO_2_ NPs	Sorafeni	Passive targeting	Reprogramming of TAMs	[263]
	Iron oxide NPs	Trastuzumab, hIgG	Passive targeting and active targeting	Depletion of TAMs	[264]
	Zinc oxide (ZnO) NPs	Doxorubicin (Dox)	Passive targeting	Reprogramming of TAMs	[265]
	Mesoporous silica-coated white-light emitting NaYbF4:Tm@NaYF4:Yb/Er upconversion NPs	Roussin’s black salt and doxorubicin	Passive targeting	Interference with TAMs survival	[266]
	NaYF4: Yb, Er@NaYF4 rare-earth upconversion-NPs conjugated with rose bengal (NPR)	Conjugated photosensitizer	Passive targeting	Reprogramming of TAMs	[249]
	Peptide/hyaluronic acid/protamine/CaCO3/DNA-NPs	pDNA and IL-12	CD44	Reprogramming of TAMs	[260,265]
	Ca-CO3/Au-NPs		Passive targeting	Reprogramming of TAMs	[227]
	Calcium-biphosphonate-PEG NPs	Chelator-free radiolabeling	Passive targeting	Depletion of TAMs	[211]
	Porous silicon multistage nanovectors (MSV)	Albumin-bound paclitaxel	Passive targeting	Reprogramming of TAMs	[267]
	MUC-1-modified AuNPs	Mucin-1 peptide	Passive targeting	Reprogramming of TAMs	[268]
Carbon NPs	Multi-walled carbon nantotubes	Fluorescent dye	Passive targeting	Reprogramming of TAMs	[222,269]
	Single-walled carbon nanotubes		Passive targeting	Depletion of TAMs	[270]
	Graphene oxide NPs	FITC and PEG	Passive targeting	Reprogramming of TAMs	[201]
	PEG and polyethylenimine (PEI) dual-polymer-functionalized graphene oxide NPs	CpG	Passive targeting	Reprogramming of TAMs	[271]
	PEG and polyethylenimine (PEI) dual-polymer-functionalized graphene oxide NPs	CpG	Passive targeting	Reprogramming of TAMs	[271]
	Polyhydroxylated fullerenols		Passive targeting	Reprogramming of TAMs	[272]
Hybrid NPs	Lipid polymer (7C1) NP	CX3CL1 siRNA	Passive targeting	Inhibition of macrophage recruitment	[217]
	Mannosylated cationic nano hydrogel particles (ManNP)	siRNA	Mannose receptor	Depletion of TAMs	[232]
	Mannose-decorated Pluronic^®^-F127 polymer and tannic acid NPs	F127-TA	Mannose receptor	Targeting TAMs	[234]
	Block copolymers with a hydrophobic block polystyrene (PS) and a hydrophilic glycoblock (PR) self-assembled into glyco-NPs with an inert organic core PS and a glycopolymer shell		Passive targeting	Reprogramming of TAMs	[244]
	Legumain-sheddable PEG5k and tuftsin dual-modified NPs	PEG5k and tuftsin	Fc receptor	Depletion of TAMs	[273]
Other	Hydroxyl dendrimer	CSF-1R inhibitor BLZ945	Passive targeting	Reprogramming of TAMs	[224]

## 5. NPs as a Delivery Platform for Gene-Editing Toolsdelivery Platform for Gene Editing Tools to Macrophagesmacrophages

The development of gene-editing tools, such as CRISPR, has opened novel avenues for the treatment of cancer. Theoretically, this revolutionary technology could bring novel therapeutic modalities to many diseases, including cancer, by precisely manipulating cellular DNA sequences. However, to date, the low efficiency of in vivo delivery and gene editing, as well as the lack of specificity towards target cells and/or tissues, must be enhanced before its therapeutic potential can be fully realized. NP-based CRISPR/Cas9 delivery systems represent a novel technology in which NPs instead of viruses are employed to directly deliver the CRISPR/Cas9 complex, or plasmids expressing Cas9 and guide RNAs, to the nuclei of targeted cells. In recent years, many NP formulations have been described as suitable non-viral delivery systems for CRISPR/Cas9, including lipid, polymeric and inorganic NPs [274]. Several molecules, such as CD47 (“do not eat me” signal), which are implicated in tumor immune escape mechanisms and can be targeted by small-molecule drugs [4], have now become targets of gene-editing approaches utilizing NPs as a delivery system for CRISPR/Cas9. Ray et al. described an integrated nanotechnology immunotherapy approach that uses arginine NPs (Arg NPs) to deliver the CRISPR/Cas9 complex into cells to generate SIRP-α knockout macrophages [254]. Knockout of SIRP-α prevented CD47:SIRP-α interactions between cancer cells and macrophages, and increased the phagocytosis of tumor cells by macrophages 4-fold. Lee et al. fabricated nanocomposites consisting of engineered Cas9 proteins that included a 20-glutamic acid tag (Cas9E20)—a strategy that has been shown to lead to the formation of hierarchical nanocomposites with NPs featuring arginine head groups (Arg NPs) through the carboxylate–guanidinium interaction [255]. The authors complexed Arg NPs, Cas9E20 and sgRNAs targeting the PTEN gene to formulate a single nanocomposite encapsulating the ribonucleoprotein complex. Systemic injection of this complex provided efficient (>8% and >4%) in vivo gene editing of the PTEN gene, specifically in macrophages of the liver and spleen, respectively. In summary, NPs as platform for gene-editing tools that specifically target TAMs open new avenues for selective immunotherapy for the treatment of cancer.

## 6. Comparative Analysis of TAM-Targeting Strategies Targeting Strategies

As discussed in Section 4.6, active targeting strategies have been widely employed to improve NPs drug delivery to TAMs. However, drug delivery to TAMs can also be achieved without linking targeting motifs to the surface of NPs [252]. In the following paragraph we will discuss advantages and disadvantages of active and passive NP-NPs targeting strategies that need to be considered when designing NPs-based therapeutics targeting TAMs.

### 6.1. Active Versus Passive Targetingversus Passive Targeting of TAMs by NPs: Advantages and Disadvantages

In contrast to the notion that targeting increases tumor localization, several studies in mice have shown that linking targeting motifs to the surface of NPs does not affect NP biodistribution or increase NP accumulation in the TME [243,275,276,277]. Rather, the dominant factors determining the accumulation of NPs in the TME are the leakiness of the vasculature and the presence of lymphatic drainage. In contrast to most normal tissues, which are characterized by tight endothelial linings that prevent the penetration of macromolecules and NPs, murine tumors possess enlarged endothelial gaps that facilitate the preferential accumulation of NPs at the tumor site. We and others have recently shown that systemically applied PEGylated PLGA NPs were able to localize to the TME in mouse models of colorectal cancer, and could be detected in different cellular subsets, in particular, myeloid cells, such as CD45+ CD11b+ F4/80+ macrophages [278]. While the importance and the existence of the EPR effect in humans is heavily debated, accumulating evidence suggests that it also occurs in human patients, but that it greatly varies between patients and type of tumor [279]; thus, tumor-targeting approaches beyond the EPR effect are equally important. Recently, it became clear that the way that NPs enter solid tumors is more complex than previously thought, and that immune cells in the TME play important roles in NP accumulation, retention and intratumoral distribution. For example, the intratumor retention of antibody-labeled NPs was shown to be determined mainly by tumor-associated dendritic cells, neutrophils, monocytes and macrophages, and not by antibody–antigen interaction [264].

Based on their phenotype, mobility and localization in the TME, different types of TAMs have been identified both in murine and human tumors. For instance, a subset of M2-like perivascular (PV) macrophages resides proximal to the abluminal surface of blood vessels and is highly efficient in sampling particular substances after intravenous injection [280]. Intravital imaging studies have shown that PV macrophages interact closely with the endothelial cells and actively remodel the permeability of neighboring blood vessels by the expression of VEGFA [281]. Due to their proximity to blood vessels and their phagocytotic nature, PV TAMs are ideally positioned to take up NPs, even in the absence of targeting motifs on the surface of NPs. Compared to non-targeted NPs, a number of active targeting approaches have resulted in decreased blood circulation times due to opsonization. For example, Salvati et al. found that transferrin-conjugated NPs placed in a biological environment were immediately coated with a protein corona, which shielded transferrin on the surface of NPs, resulting in a marked reduction in binding specificity [174]. Thus, the global delivery of drugs to the TME and TAMs can be efficiently achieved by passive targeting. This strategy might in particular be suited for the delivery of membrane- permeable drugs, such as cyclodextrins, which upon NPs accumulation in the TME diffuse from the core of NPs and are taken up by surrounding cells [282].

As outlined above, passive targeting circumvents some drawbacks of targeted NPs approaches, however, the delivery of NPs to target cells by the recognition of surface receptors also shows clear advantages. First, specific payload delivery to target cells reduces drug toxicity and keeps adverse immune reactions at minimum. Second, active targeting strategies have demonstrated increased NPs internalization inside target cells and intracellular drug delivery, compared to non-targeted NPs. Thus, the active targeting approach is in particular suited for the cell-specific delivery of membrane- impermeable drugs, and/or co-delivery of compounds, such as guide RNA and Cas9 [283], to specific cell types in vivo. For example, DSPE-PEG-EGFR-siRNA NPs were coated with the ligand anisamide to target sigma receptors and administered intravenouslyintravenous in mice bearing a sigma receptor expressing NCI-H460 xenograft tumors [275]. While the targeting ligand did not influence the distribution of the NPs, it increased the intratumoral localization of the siRNA and silencing of EGFR expression. The targeted NPs formulation efficiently delivered siRNA into the cytoplasm of NCI-H460 tumor cells, whereas free siRNA and non-targeted NPs showed little uptake. Third, targeting specific cell types instead of global administration might overcome limitations of some EPR-based therapies. For instance, passive drug delivery strategies based on the EPR effect are only applicable to solid tumors above a certain size [284], whereas active targeting of cell populations provides the possibility of drug delivery to a variety of tumor and non-tumor tissues. Fourth, targeting NPs via surface receptors to myeloid cells has been shown to increase adjuvanticity and immunogenicity in immunotherapy [14,18,285,286].

Active targeting provides the possibility to target TAM subsets. In this context, it has been shown that spatially distinct TAM subsets with different phenotypes and behavior co-exist in the TME. The spatial heterogeneity is most likely as an adaptation to the different tumor compartments, such as the tumor nest, stroma and PV niche, and TAM subsets show differential expression of cell surface (such as angiopoietin-1 receptor, MR, CD163, TLR4, PD-L1. CD100, CD80 and CD86) and intracellular/secreted proteins (cathepsins, urokinase-type plasminogen activator, nitric oxide synthase 2, pro-MMP7, VEGFA, pro-MMP10 and IL-10). Evidence is emerging that TAM subsets in certain tumor areas limit tumor responses to treatment. For example, PV TAMs have been implicated in contributing to relapse after chemotherapy, and hypoxic TAMs in the tumor resistance to anticancer therapy [287]. Thus, active targeting by NPs could provide the tool to deplete or re-educate those TAM subsets that are tumor-promoting, while keeping those TAMs that are tumoricidal and/or promote antitumor immunity.

### 6.2. Comparison of NP-Based TAM-Targeting Therapies and Current Challenges

Current NP-based TAM-targeting strategies have shown great potential in preclinical and clinical cancer research, and can greatly improve therapeutic effects while reducing systemic side effects [288]. NPs that treat TAMs in cancer therapy have been designed to inhibit TAM recruitment or accumulation, or to re-educate TAMs to restore functional phagocytosis and M1 phenotype. In the following paragraph we compare the most common TAM-targeting approaches utilizing NPs and discuss associated challenges. TAM depletion is often achieved by administration of drug-loaded NPs (Table 2); however, administration of non-targeted drug-loaded NPs can evoke immune toxicity through undesired NP interaction with immune cells; thus, in-depth toxicity studies must be carried out to carefully evaluate potential off-target effects. For example, acute toxicity has been observed when administering liposomal zoledronic acid, most likely due to increased cytokine production [289]. In addition, NPs with immunomodulatory functions should be designed to target specific cells to limit off-target effects and to provide a sustained antitumor effect in patients. The long-term consequences of TAM depletion are still unclear; therefore, it might not be desired to eliminate both proinflammatory and anti-inflammatory types of TAMs. Rather, subset-specific targeting ligands are needed to deplete or re-educate those that are tumor-promoting, while leaving or increasing those that promote antitumor immunity. Another drawback of this method is that it is likely some TAMs will remain after treatment which could drive tumor relapse [43]. Mannose and galactose are often used as a targeting ligand for NP-mediated drug delivery to TAMs (Table 2); however, mannose/galactose receptors are present on both M1- and M2-type TAMs. In addition, TAMs often display intermediate states (in-between M1 and M2 phenotypes) and other cell types may display these receptors as well [290,291]. Therefore, targeting specificity remains a key challenge. Thus, ligands that are more specific to M2 TAMs, such as M2pep [245,246], might represent better targeting moieties for NP-mediated drug delivery to TAMs. A lack of specificity might lead to the undesired targeting of tissue-resident macrophages, such as red pulp macrophages in the spleen or Kupffer cells in the liver. Another strategy focuses on the depletion of TAMs or inhibition of TAM recruitment, which is often achieved by the delivery of molecules (e.g., siRNA) that inhibit trafficking receptors, such as CCR2. However, this strategy does not take into account tissue-resident macrophages that do not seem to be recruited in the same way as monocyte-derived macrophages. Thus, strategies that can differentially block the recruitment and proliferation of tissue-resident macrophages are needed. Finally, most NP formulations constructed to treat TAMs focus on the modulation of the polarization status of TAMs towards an antitumorigenic phenotype. However, more research is needed to evaluate the long-term effect of this approach. Excessive macrophage activation might lead to substantial cell-mediated toxicity, such as macrophage activation syndrome and hemophagocytic syndrome.

Therapeutic approaches to deplete or reprogram TAMs, including inhibitors targeting the CSF-1-CSF-1R and the CCL-CCR2 axes, have largely failed to show efficacy in cancer clinical trials as monotherapies [292,293]. One explanation could be that the inhibition of one component might be compensated by another. For this reason, multimodal treatment approaches are expected to improve the outcome of immunotherapies in different types of cancer. NPs carrying different drugs for combination therapy have been shown to improve the therapeutic efficacy and to overcome multidrug resistance [252,294]. For example, a humanized anti-SEMA4D antibody (pepinemab) is currently being tested in multiple clinical trials for various cancers (NCT03320330, NCT03690986 and NCT03769155), in combination with PD1/PDL1 checkpoint inhibitors [295]. M2 -TAMs produce SEMA4D, which among other effects has been reported to inhibit immune cell movement and to cause vascular destabilization. Combinatorial treatment strategies targeting these two pathways in a synergistic manner are expected to have a greater impact on the inhibition of tumor growth, than PD1/PDL1 checkpoint inhibitors or anti- SEMA4D monotherapies. The combination of TAM-targeted drugs with chemotherapy could also improve the therapeutic effect of chemotherapeutic drugs, which when administered alone can increase the infiltration of TAMs into tumor tissues [258,296]. Furthermore, the combination of M2- TAM-targeting by Au@SiO2 (GNPs) NPs conjugated with a CD163 antibody with radiation has resulted in more effective tumor growth delay than radiation alone [259]. Synergistic effects were also observed when gadofullerene NPs were combined with anti-PD-L1 immune checkpoint inhibition [259]. Gadofullerene NPs were shown to reprogram TAMs to a M1-like phenotype and increase the infiltration of cytotoxic T lymphocytes. Combinational treatment with anti-PD-L1 achieved effective inhibition of tumor growth in the 4T1 murine breast cancer model in vivo.

## 7. Conclusions and Future Outlook

As described in this review, the TME plays an important role in tumorigenesis. As one of the main cell populations of tumor-infiltrating immune cells, macrophages play a central role in homeostasis in inflammatory diseases and immunotherapy.

More recently, TAMs have emerged as a target of NPs-mediated cancer immunotherapy. The internalization mechanism of targeted NPs in macrophages and their capacity to repolarize the TAMs phenotype of TAMs in cancer treatment have received increasing attention. More and more evidence shows that eliminating or manipulating TAMs is beneficial for cancer treatment. The inhibition of macrophage CSF1R activity or the enhancement of STAT3 activation in the TME can promote the repolarization of M2 macrophages to a phenotype similar to M1 to overcome the immunosuppressive state that promotes tumor growth and metastasis. However, pan-macrophage therapy targeting all macrophages will cause systemic toxicity. Therefore, immunotherapy strategies specifically targeting immunosuppressive TAMs in the TME by exploiting TAM-specific cell surface receptors, combined with immunotherapy, provide new opportunities for tumor diagnosis and treatment.

Because certain characteristics of in vitro and in vivo macrophages are not completely consistent, multi-level verification in animal models is required, as outlined in this review. In vivo, NPs can be passively targeted through the EPR effect or actively targeted to tumors through conjugated ligands for specific drug delivery. The physicochemical properties of NPs (size, shape, surface charge and, targeting moieties) affect NPs uptake in different TAM subsets and consequently determine the NP efficacy. Thus, NPs preparation and the use of targeting moieties are important points to consider when designing NPs to target TAMs. Additionally, the choice of payload, such as drug and adjuvants, will require thorough consideration when designing novel NP-based treatments with low systemic toxicity. Thus, to design more efficient delivery systems that specifically target TAMs and to improve the concentration of drugs at the tumor site in the course of immunotherapy.

To overcome some limitations of current TAM-targeting NP formulations, such as off-target effects, stimuli-responsive NPs that selectively release therapeutic agents in target tissues or cells have been designed. Such NP systems with spatio-temporal release kinetics can release therapeutics “on-demand” in a controlled fashion, and are often combined with molecular imaging to monitor the biodistribution or activation of these systems. Examples of such a system are NPs that are built from materials that “respond” to intrinsic stimuli, such as pH, enzymes and reducing agents, as well as extrinsic stimuli such as heat, light, electric fields, ultrasound and magnetic fields [297]. For example, Liang and coworkers recently developed a novel dually functionalized NP platform by the surface co-modification of NPs with tuftsin, a natural activator of phago-cytosis, and legumain-protease-sheddable PEG to achieve selective targeted delivery to TAMs [273]. In another example, MMP2-sensitive phosphatidylserine-modified NPs loaded with the model drug dasatinib were developed [256]. In this design, the phosphatidylserine was externalized to the NPs’ surface only when the NPs reached the MMP2-overexpressing TME, allowing for the TAM-specific phagocytosis. The NPs showed excellent specificity towards TAMs in various biological models. Importantly, the specificity of NPs towards TAMs remarkably enhanced TAM depletion. Overall, such novel NPs platforms with an “on-demand” release kinetics design could greatly improve the selectivity and efficacy of targeted NP therapeutics towards TAMs.

## Data Availability

No new data were created or analyzed in this study. Data sharing is not applicable to this article.
